# Grain Boundary‐Derived Cu^+^/Cu^0^ Interfaces in CuO Nanosheets for Low Overpotential Carbon Dioxide Electroreduction to Ethylene

**DOI:** 10.1002/advs.202200454

**Published:** 2022-05-22

**Authors:** Jianfang Zhang, Yan Wang, Zhengyuan Li, Shuai Xia, Rui Cai, Lu Ma, Tianyu Zhang, Josh Ackley, Shize Yang, Yucheng Wu, Jingjie Wu

**Affiliations:** ^1^ School of Materials Science and Engineering Hefei University of Technology Hefei 230009 China; ^2^ Department of Chemical and Environmental Engineering University of Cincinnati Cincinnati OH 45221 USA; ^3^ National Synchrotron Light Source II Brookhaven National Laboratory Upton NY 11971 USA; ^4^ Eyring Materials Center Arizona State University 85287 Tempe AZ USA; ^5^ China International S&T Cooperation Base for Advanced Energy and Environmental Materials and Anhui Provincial International S&T Cooperation Base for Advanced Energy Materials Hefei University of Technology Hefei 230009 China

**Keywords:** CO_2_ reduction, Cu^+^/Cu^0^ interfaces, ethylene, grain boundary, low overpotential

## Abstract

Electrochemical CO_2_ reduction reaction can be used to produce value‐added hydrocarbon fuels and chemicals by coupling with clean electrical energy. However, highly active, selective, and energy‐efficient CO_2_ conversion to multicarbon hydrocarbons, such as C_2_H_4_, remains challenging because of the lack of efficient catalysts. Herein, an ultrasonication‐assisted electrodeposition strategy to synthesize CuO nanosheets for low‐overpotential CO_2_ electroreduction to C_2_H_4_ is reported. A high C_2_H_4_ Faradaic efficiency of 62.5% is achieved over the CuO nanosheets at a small potential of −0.52 V versus a reversible hydrogen electrode, corresponding to a record high half‐cell cathodic energy efficiency of 41%. The selectivity toward C_2_H_4_ is maintained for over 60 h of continuous operation. The CuO nanosheets are prone to in situ restructuring during CO_2_ reduction, forming abundant grain boundaries (GBs). Stable Cu^+^/Cu^0^ interfaces are derived from the low‐coordinated Cu atoms in the reconstructed GB regions and act as highly active sites for CO_2_ reduction at low overpotentials. In situ Raman spectroscopic analysis and density functional theory computation reveal that the Cu^+^/Cu^0^ interfaces offer high *CO surface coverage and lower the activation energy barrier for *CO dimerization, which, in synergy, facilitates CO_2_ reduction to C_2_H_4_ at low overpotentials.

## Graphical abstract

Abundant grain boundaries involving stable Cu^+^/Cu^0^ interfaces are in situ reconstructed in the CuO nanosheets during CO_2_ electroreduction. The reconstructed grain boundaries with Cu^+^/Cu^0^ interfaces increase the *CO surface coverage and lower the energy barrier for *CO dimerization, leading to a record maximal half‐cell cathodic energy efficiency of 41% for C_2_H_4_ formation.

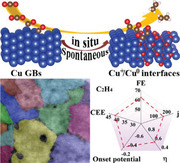

## 1 Introduction

Electrochemical CO_2_ reduction reaction (eCO_2_RR) can be employed to produce value‐added hydrocarbon chemicals (e.g., ethylene, C_2_H_4_) by coupling with clean electrical energy.^[^ ^1^, ^2^ ^]^ According to recent technoeconomic assessment studies, the production cost of C_2_H_4_ in the eCO_2_RR process is estimated to be as high as US$2.48 per kg C_2_H_4_; thus, it is not economically beneficial, as the market price is US$0.55 per kg C_2_H_4_.^[^ ^3^ ^]^ Increasing the energy efficiency of a CO_2_ electrolyzer is a feasible approach to reducing production costs. Cu catalysts have achieved significant advances in the production rate and Faradaic efficiency (FE) of C_2_H_4_.^[^ ^4^, ^5^ ^]^ However, they still suffer from low energy efficiency as the peak FE of C_2_H_4_ appears at considerably negative cathodic potentials from −0.7 to −1.0 V (vs reversible hydrogen electron (RHE hereafter)).^[^ ^4^, ^6^ ^]^ Reducing the overpotential of the eCO_2_RR over Cu catalysts while maintaining the selectivity and productivity toward a target C_2+_ product can improve the market competitiveness of the eCO_2_RR; however, this remains a big challenge.

Oxide‐derived Cu (OD‐Cu) catalysts exhibit a significantly lower overpotential of the eCO_2_RR toward C_2+_ products than Cu metal catalysts.^[^ ^7^, ^8^, ^9^, ^10^, ^11^ ^]^ The two main features of the OD‐Cu catalyst, including defect sites with low‐coordinated Cu atoms and polarized Cu^*δ*+^ (0 < *δ* ≤ 1) induced by residual subsurface O, have been deemed responsible for promoting the C—C coupling step at low overpotentials.^[^ ^11^, ^12^, ^13^, ^14^, ^15^, ^16^ ^]^ The defect sites combining the strain and low coordination number effects enhance *CO adsorption, increasing the CO coverage of the catalyst, which kinetically favors C—C coupling over hydrogenation.^[^ ^17^ ^]^ Similarly, both the experimental results and theoretical calculations provide solid evidence that *CO adsorption on the Cu^+^ site is stronger than that on the Cu^0^ site because of the unoccupied 3d orbital of Cu^+^.^[^ ^18^, ^19^ ^]^ Moreover, Cu^+^/Cu^0^ interfaces promote CO_2_ activation and CO dimerization.^[^ ^10^, ^20^, ^21^, ^22^ ^]^ The Cu^+^/Cu^0^ interface has more favorable energetics for C—C coupling than a single Cu^+^ or Cu^0^ site because attractive electrostatic interactions between the two Cs of CO assist the C—C bond formation.^[^ ^10^ ^]^ However, the existence of subsurface O during the eCO_2_RR is a long‐standing debate in literature because its presence depends on the history of the material.^[^ ^16^, ^23^, ^24^, ^25^, ^26^ ^]^ Further, the theoretical prediction of the stability of subsurface O differs because of the difference in the selection of the material model.^[^ ^27^, ^28^, ^29^, ^30^, ^31^ ^]^ Thermodynamically, the CuO will be fully reduced at neutral and alkaline pH for applied potentials lower than −0.1 V.^[^ ^32^ ^]^ However, the grand canonical potential kinetics density functional theory method has predicted that subsurface O is preserved in the disordered Cu region with low coordination.^[^ ^29^, ^30^, ^31^ ^]^ Notably, the electrochemical standard redox potentials of Cu^*δ*+^/Cu^0^ shift to a more negative value with the decrease in the bulk size to a few atoms (e.g., −2.7 V for Cu^+^/Cu° for a single Cu atom).^[^ ^33^ ^]^ This suggests that the Cu^*δ*+^ species with the subsurface O can be thermodynamically trapped in the grain boundaries (GBs) containing abundant low‐coordinated Cu atoms. Therefore, it is reasonable to assume that stable Cu^*δ*+^ species can induce active Cu^*δ*+^/Cu^0^ interfaces across Cu GBs.

The GBs in the OD‐Cu are formed by the in situ reduction and reconstruction of CuO nanostructures during the eCO_2_RR.^[^ ^34^, ^35^, ^36^ ^]^ The electrochemical reduction of CuO or Cu_2_O to Cu can create more lattice distortions and defects, which decrease the structural stability by improving the surface energy of the Cu atoms.^[^ ^34^, ^35^ ^]^ The undercoordinated Cu atoms around the lattice distortions and defect regions are conventionally unstable and inclined toward surface reconstruction to form small‐sized grains.^[^ ^37^ ^]^ A nanoclustering process is proposed to form relatively small Cu nanoclusters and nanoparticles with an increasing number of low‐coordinated sites during the eCO_2_RR.^[^ ^38^ ^]^ Resultantly, large CuO or Cu_2_O nanoparticles spontaneously evolve into small Cu nanograins with abundant GBs during dynamic reconstruction. The dislocated lattices with low‐coordinated Cu atoms in the reconstructed Cu GBs provide defect sites for oxide nucleation, affording Cu^*δ*+^ species along the Cu GBs.^[^ ^39^ ^]^

Here, we report a low overpotential yet highly active and selective CO_2_‐to‐C_2_H_4_ reduction over GBs involving Cu^+^/Cu^0^ interfaces. Rich‐density GBs are formed via in situ reduction of CuO nanosheets and subsequent reconstruction during the eCO_2_RR. An ultrasonication‐assisted electrodeposition method is developed for the facile synthesis of CuO nanosheets. In contrast to the electrodeposition onto a substrate in the conventional process, ultrasonication‐assisted electrodeposition attains freestanding CuO nanosheets formed by peeling Cu nanosheets off the substrate by ultrasonication, followed by the spontaneous oxidation of Cu in an alkaline solution. To the best of our knowledge, the prepared CuO nanosheets exhibit the lowest potential of −0.52 V to achieve an industrially relevant C_2_H_4_ partial current density $\left(j_{C_{2}H_{4}}\right)$ of 173 mA cm^−2^ in the eCO_2_RR. A high FE of C_2_H_4_ ($FE_{C_{2}H_{4}}$) of 62.5% at such a low potential contributes to a maximal half‐cell cathodic energy efficiency (CEE) of 41%. The low‐coordinated Cu atoms in the GB regions shift the electrochemical redox potential to more negative values, thereby stabilizing the Cu^+^ species and creating the Cu^+^/Cu^0^ interfaces across the GB width. The combined results of in situ Raman spectra and density functional theory (DFT) computations suggest that the reconstructed GBs with the Cu^+^/Cu^0^ interfaces serve as primary active sites that steer the C—C coupling to produce C_2_H_4_ at low overpotentials along the pathway of the *CO dimerization.

## 2 Results and Discussion

### 2.1 CuO Nanosheets Synthesis and Characterization

**Figure 1:**
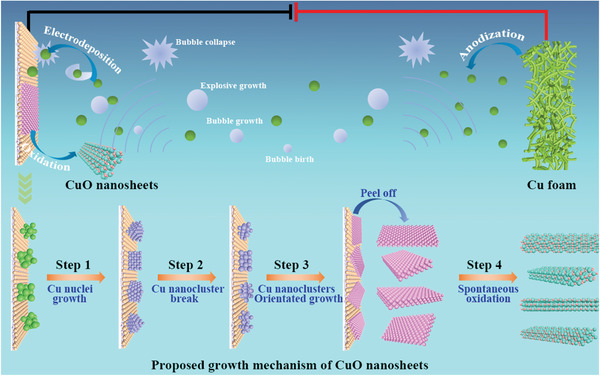
Schematic illustration of ultrasonication‐assisted electrodeposition of CuO nanosheets.

An ultrasonication‐assisted electrodeposition method was developed to synthesize the CuO nanosheets at a large scale. Briefly, the synthesis of the CuO nanosheets was performed in a galvanostatic mode in an undivided two‐electrode cell equipped with a Cu‐foam anode and carbon‐paper cathode in a 3 m KOH electrolyte solution, as shown in **Figure** **1**. The electrodeposition cell was placed in an ultrasonic bath at an ultrasonic power of 160 W. The entire electrodeposition process was reflected by chronopotentiometry *E*–*t* curves (Figure S1, Supporting Information). First, Cu^2+^ ions were produced by the electrochemical anodization of the Cu‐foam at the anode, and the ions were coordinated with OH^−^ ions to form [Cu(OH)_4_]^2−^ complexes, followed by reduction to Cu on the carbon‐paper cathode (stage I). Subsequently, the gradually cumulative Cu‐nanosheet electrodeposits on the cathode were peeled off under ultrasonication and oxidized into CuO nanosheets in the KOH electrolyte (stage II). Owing to the accumulation of Cu(OH)_2_ on the surface of the Cu foam, the predominant reaction on the anode was switched from Cu anodization to the O evolution reaction (OER) on Cu(OH)_2_ during electrodeposition for 40 min. The applied potential abruptly increased because of the activation polarization caused by the sluggish kinetics of the OER. The real‐time captured photo images of the electrolyte revealed that this proposed two‐stage reaction process produced large quantities of CuO nanosheets (Figure S2, Supporting Information). The synthetic process of the CuO samples at relatively low ultrasonic powers (0–80 W) exhibited similar *E*–*t* curves (Figure S1, Supporting Information). However, the periods of Cu anodization were shortened to 20 min at 80 W and further to 5 min at 0 W. These results indicated that the ultrasonic power could regulate the anodic reaction time for Cu anodization versus the OER. A high ultrasonic power corresponded to the anodic reaction duration for Cu anodization to form Cu^2+^ ions, increasing the yield of CuO nanosheets by cathodic electrodeposition.

**Figure 2:**
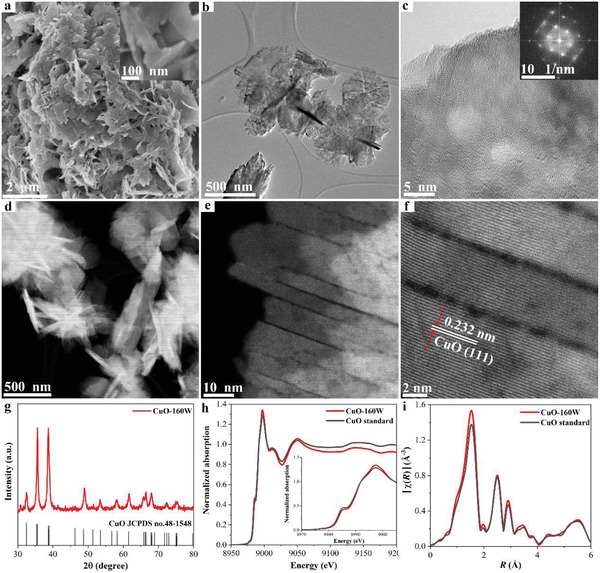
Structural characterization of CuO nanosheets. a) SEM image, b) TEM image, c) high‐resolution TEM image and corresponding FFT pattern, d) HAADF‐STEM image, e,f) high‐resolution STEM image, g) XRD pattern, h) Cu K‐edge XANES spectrum, and i) Cu K‐edge Fourier transformed EXAFS spectrum.

When prepared at a constant electrodeposition current density of 190 mA cm^−2^ under an ultrasonic power of 160 W, the CuO showed a nanosheet morphology, as shown by scanning electron microscopy (SEM, **Figure** **2**a) and transmission electron microscopy (TEM, Figure 2b) images. High‐resolution TEM (HRTEM) images and the corresponding fast Fourier transform (FFT) revealed an integral crystal plane with a single orientation of the (111) face (Figure 2c,f). The ultrasonic process facilitated the formation of the CuO nanosheets and introduced abundant cracks into the CuO nanosheets, as illustrated by the high‐angle annular dark‐field scanning transmission electron microscopy (HAADF‐STEM) images shown in Figure 2d–f. X‐ray diffraction (XRD) analysis provided evidence of a pure CuO phase in the as‐prepared samples (Figure 2g). The normalized X‐ray absorption near edge structure (XANES, Figure 2h) of the CuO nanosheets suggested the presence of only Cu^2+^ species. The coordination environment of the Cu atom in the CuO nanosheets is the same as that of the standard CuO reference, as identified by the Fourier‐transformed extended X‐ray absorption fine structure (EXAFS, Figure 2i), further demonstrating the pure composition of the CuO nanosheets. The combination of the X‐ray photoelectron spectroscopy (XPS) and Auger spectroscopy results of the Cu 2p, O 1s, and Cu LMM spectra confirmed that only Cu^2+^ species were present in the CuO nanosheets (Figure S3, Supporting Information).

We systematically investigated the effects of the synthetic parameters on the formation of CuO nanosheets. First, ultrasonic waves played a critical role in the formation of CuO nanosheets. Without ultrasonication (ultrasonic power of 0 W), the Cu sample electrodeposited on the carbon paper showed a microsphere structure composed of nanoparticles at a deposition current density of 190 mA cm^−2^ (Figure S4, Supporting Information). The as‐deposited Cu could be oxidized to CuO (denoted as CuO‐0W) in KOH solution when the applied current was stopped, as confirmed by the XRD results (Figure S5, Supporting Information). The microspheres continuously deposited on the carbon paper without falling off into the solution during the 1 h electrodeposition. By contrast, the accumulated Cu samples were peeled off from the carbon paper assisted by the ultrasonic wave and oxidized to CuO in the KOH solution, as demonstrated by the XRD (Figure S6, Supporting Information). At a low ultrasonic power of 80 W, the obtained CuO (CuO‐80W) presented a hybrid morphology of nanosheets and nanoparticles, indicating that some partially cracked Cu nanoclusters aggregated to form nanoparticles at a relatively low ultrasonic power (Figure S4, Supporting Information). The fragmented Cu nanoclusters were fully transformed into CuO nanosheets when the ultrasonic power was increased to 160 W (CuO‐160 W). Second, the electrodeposition current density controlled the Cu electrodeposition rate, thereby affecting the morphology of CuO. Five CuO‐160W samples were synthesized using different electrodeposition current densities from 160 to 220 mA cm^−2^ under a fixed ultrasonic power of 160 W. CuO showed an irregular shape containing thick sheets at 160 mA cm^−2^ (Figure S7, Supporting Information). The morphology of CuO changed to nanoflower, which was an assembly of nanosheets, when the current density increased to 180 mA cm^−2^ and evolved into distinguishably thin nanosheets as the current density increased to 190 and 200 mA cm^−2^. A further increase in the current density to 220 mA cm^−2^ resulted in a dendritic structure accompanied by a small fraction of nanosheets.

Based on the above results, we propose a four‐step growth mechanism for the CuO nanosheets, as shown in Figure 1. Cu nuclei were rapidly produced by the electrochemical reduction of the [Cu(OH)_4_]^2−^ complexes near the carbon‐paper cathode and continuously grown into Cu nanoclusters upon the reduction of more [Cu(OH)_4_]^2−^ complexes (step 1). The ultrasonication induced the cavitation effect, which provided vacuum bubble reaction conditions with extremely high temperatures and pressures.^[^ ^40^ ^]^ The bubbles were born and grown under ultrasonication and finally collapsed, facilitating the symmetrical breaking of the Cu nanoclusters (step 2). Resultantly, the cracked Cu nanoclusters were oriented toward the growth of the nanosheet structures attached to the cathode (step 3). Small nanosheets with adherent nanoparticles were observed on the electrodeposited Cu on the carbon paper (Figure S8, Supporting Information), verifying the growth process of the Cu nanosheets. Ultimately, the accumulated Cu nanosheets were peeled off from the cathode under ultrasonication and oxidized into CuO nanosheets in the KOH solution (step 4).

### 2.2 CO_2_ Electroreduction Performance

**Figure 3:**
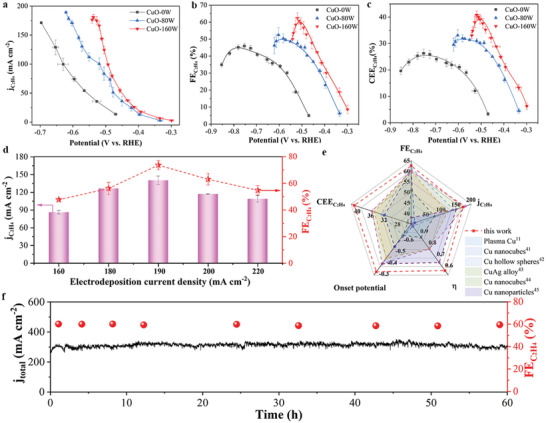
Electrochemical performance of eCO_2_RR. a) Partial current densities, b) faradaic efficiencies, c) cathodic energy efficiencies of C_2_H_4_ on CuO‐0W, CuO‐80W, and CuO‐160W under various potentials, d) compared faradaic efficiencies and partial current densities of C_2_H_4_ among various CuO‐160W electrodes prepared by different electrodeposition current density, e) comparison of partial current density, FE, half‐cell CEE, onset potential and overpotential (*η*) of C_2_H_4_ on CuO‐160W with those of state‐of‐the‐art Cu‐based catalysts, and f) long‐term stability test of CuO‐160W at −0.52 V (black line, total current density; red ball, C_2_H_4_ faradaic efficiency).

The effect of ultrasonic power on the CuO‐based electrodes on the eCO_2_RR was evaluated in a liquid electrolyte flow cell using a 1 m KOH aqueous electrolyte. We compared the activity and selectivity of the eCO_2_RR among CuO‐0W, CuO‐80W, and CuO‐160W prepared at the same electrodeposition current density of 190 mA cm^−2^. The CuO‐0W electrode exhibited the most negative onset potential of −0.47 V for the formation of C_2_H_4_ (**Figure** **3**a). The onset potential shifted positively to −0.33 V for the CuO‐80W electrode and further down to −0.28 V for the CuO‐160W electrode. The $j_{C_{2}H_{4}}$ dramatically increased in the order of CuO‐0W < CuO‐80W < CuO‐160W electrodes in the potential range from −0.28 to −0.55 V (Figure 3a; Figure S9, Supporting Information). In particular, the CuO‐160W electrode achieved a high $j_{C_{2}H_{4}}$ of 173 mA cm^−2^ at a low cathodic potential of −0.52 V, outperforming prior Cu‐based catalysts (Table S1, Supporting Information). The surface roughness or electrochemical active surface area (ECSA) of these three electrodes was estimated by calculating the double‐layer capacitance (*C*_dl_) from cyclic voltammetry curves at various scan rates (Figure S10, Supporting Information). The CuO‐160W electrode showed considerably high ECSA‐normalized $j_{C_{2}H_{4}}$ (Figure S11, Supporting Information), indicating that the surface roughness could not fully account for the increased activity and selectivity of the eCO_2_RR on the CuO‐160W electrode. Figure S12 of the Supporting Information shows the FEs of all the eCO_2_RR products as a function of the applied potentials for the CuO‐0W, CuO‐80W, and CuO‐160W electrodes. The CuO‐0W electrode required more negative potentials to reach a moderate $FE_{C_{2}H_{4}}$, e.g., a peak $FE_{C_{2}H_{4}}$ of 45.9% was observed at an applied potential of −0.75 V (Figure 3b). The CuO‐80W electrode showed enhanced C_2_H_4_ selectivity with a top $FE_{C_{2}H_{4}}$ of 52.4% at a considerably positive potential of −0.60 V. Interestingly, a maximum $FE_{C_{2}H_{4}}$ of 62.5% was achieved for the CuO‐160W electrode at a relatively low potential of −0.52 V. The relatively low overpotentials and relatively high $FE_{C_{2}H_{4}}$ afforded a relatively high energy efficiency for CO_2_‐to‐C_2_H_4_ conversion over the CuO‐160W electrode compared to other CuO electrode counterparts. The CuO‐160W electrode attained a maximum half‐cell CEE of 41% for C_2_H_4_ formation, which was 1.4 and 3.4 times that for the CuO‐80W (30%) and CuO‐0W (12%) electrodes, respectively (Figure 3c). These results strongly suggested the ultrasonic power dependence of the reactivity of the CuO electrodes.

Next, we analyzed the effect of the electrodeposition current density on the performance of the CuO electrodes for the eCO_2_RR. The FEs and corresponding partial current densities for the various products are shown in Figure 3d and Figures S13 and S14 (Supporting Information). For the CuO electrodes prepared at 160 W, both $FE_{C_{2}H_{4}}$ and $j_{C_{2}H_{4}}$ showed volcano profiles, and the highest was observed at the electrodeposition current density of 190 mA cm^−2^. The corresponding potential of the peak $FE_{C_{2}H_{4}}$ displayed an inverse volcano plot as a function of the electrodeposition current density. Figure 3e compares the critical performance metrics of CO_2_ electrolysis, including the onset potential, peak $FE_{C_{2}H_{4}}$, overpotential (*η*), and $j_{C_{2}H_{4}}$ for the peak $FE_{C_{2}H_{4}}$, and the corresponding half‐cell CEE between the CuO‐160W (deposited at 190 mA cm^−2^) and previous Cu‐based electrodes.^[^ ^11^, ^41^, ^42^, ^43^, ^44^, ^45^ ^]^ The CuO‐160W electrode possessed the lowest onset potential for C_2_H_4_ production. Moreover, the CuO‐160W electrode yielded the highest $j_{C_{2}H_{4}}$ at the lowest corresponding potential of −0.52 V. Accordingly, the CuO‐160W electrode attained the highest half‐cell CEE of 41%. Additionally, the long‐term stability test demonstrated that the CuO‐160W electrode maintained a steady *j*_total_ of 300 mA cm^−2^ and a stable $FE_{C_{2}H_{4}}$ of ≈60% for at least 60 h when CO_2_ electrolysis was performed at a constant potential of −0.52 V (Figure 3f).

### 2.3 Surface Reconstruction during the CO_2_ Reduction

**Figure 4:**
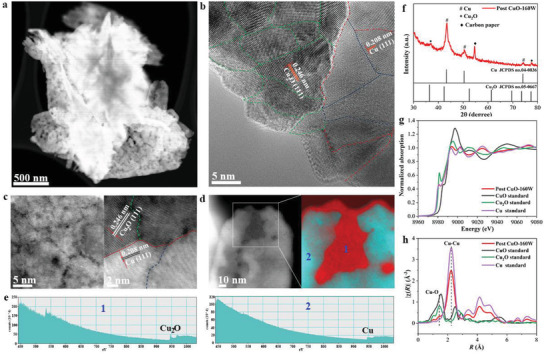
Morphological and compositional analysis of post CuO‐160W after eCO_2_RR. a) HAADF‐STEM image, b) high‐resolution TEM image, c) high‐resolution HAADF‐STEM image with magnified region of GBs and Cu^+^/Cu^0^ interfaces. Green, blue, and red lines in the right‐hand image represent Cu_2_O GBs, Cu GBs and Cu^+^/Cu^0^ interfaces, respectively. d) EELS mapping of Cu^+^ in 1 and Cu^0^ in 2, e) Corresponding EELS spectra collected from the areas of 1 and 2 in (d), f) XRD patterns, g) Cu K‐edge XANES spectra, h) Cu K‐edge Fourier transformed EXAFS spectra.

To explore the underlying mechanisms of the enhanced activity and selectivity toward CO_2_‐to‐C_2_H_4_ conversion on the CuO‐160W electrode, we first examined the morphology and chemical composition of this electrode after the eCO_2_RR by ex situ characterization analysis. The overall nanosheet structure of the CuO‐160W remained intact after the eCO_2_RR (Figures S15 and S16, Supporting Information). However, a large number of pore defects were generated on the post‐CuO‐160W nanosheets, which were observed in low‐magnification HAADF‐STEM images (**Figure** **4**a). Additionally, the HRTEM images provided direct evidence for the reconstruction of the post‐CuO‐160W nanosheets. The integral CuO(111) nanosheets were reduced and reconstructed to form small fragments of Cu_2_O(111) (outlined in green) and Cu(111) (outlined in blue), resulting in high‐density GBs (Figure 4b). The Cu_2_O grains were supposed to be formed by the reoxidation of the Cu grains. The abundant defects in the Cu GBs not only reduced the nucleation energy barrier of Cu_2_O but also improved the dissociation of O_2_ to O owing to the high O‐sticking coefficient at the defective active site.^[^ ^46^ ^]^ The high‐density Cu GBs were the preferential nucleation sites for the growth of CuO and provided channels for O diffusion, leading to the easy reoxidization of metallic Cu to Cu_2_O.^[^ ^39^, ^47^, ^48^ ^]^ Resultantly, the fragmented Cu_2_O(111) and Cu(111) grains afforded abundant Cu_2_O(111)/Cu(111) (Cu^+^/Cu^0^) interfaces (outlined in red) in the post‐CuO‐160W nanosheets. The high‐resolution HAADF‐STEM image shown in Figure 4c further exhibits both GBs and Cu^+^/Cu^0^ interfaces in the fragmented post‐CuO‐160W nanosheets. Electron energy loss spectroscopy (EELS) mapping showed the distinguished Cu_2_O and Cu grains (Figure 4d,e; Figure S17, Supporting Information). Notably, the post‐CuO‐0W microspheres showed an integrated lattice plane of Cu(111) in the HRTEM image after the eCO_2_RR (Figure S18a, Supporting Information), indicating that it was challenging to reconstruct the GBs. The post‐CuO‐80W exhibited a medium density of GBs between the Cu(111) grains (Figure S18b, Supporting Information). The post‐CuO‐160W nanosheets electrode exhibited the highest density of GBs (Figure S18c, Supporting Information). The rich density of the reconstructed GBs afforded the improved performance of the CuO‐160W nanosheets rather than the exposed (111) facet. The XRD pattern showed that the post‐CuO‐160W nanosheets were composed predominantly of Cu with minor Cu_2_O phases (Figure 4f). The Cu K‐edge XANES spectra of the post‐CuO‐160W nanosheets displayed a typical absorption edge position for Cu (Figure 4g), suggesting that metallic Cu predominated in this electrode. The corresponding EXAFS spectra showed two main peaks corresponding to the Cu—O and Cu—Cu coordination shells at 1.44 and 2.23 Å, respectively (Figure 4h). The XPS analysis of the Cu 2p, O 1s, and Cu LMM spectra further demonstrated that the surface composition of the post‐CuO‐160W nanosheets was mainly composed of Cu and Cu_2_O (Figure S19, Supporting Information).

Notably, the electrochemical standard potentials for small metal clusters containing n atoms (*n* < 20) were more negative than the value of the bulk metal (*n* >> 20).^[^ ^49^, ^50^, ^51^, ^52^ ^]^ In a limiting case, the electrochemical standard reduction potentials for a single metal atom were several volts more negative than for bulk metal (e.g., −1.5 V for Au^+^/Au_1_^0^, −1.8 V Ag^+^/Ag_1_^0^, and −2.7 V for Cu^+^/Cu_1_^0^).^[^ ^33^, ^53^, ^54^ ^]^ Lattice deformation and dislocation in reconstructed GBs created many Cu and/or Cu^+^ surface sites with a low coordination number. Thus, although the Cu_2_O(111) grains will likely be reduced to Cu^0^ during eCO_2_RR, the Cu_2_O surface sites in the region of the GBs could maintain a Cu^+^ state because they held more negative reduction potentials than the applied potential (the most negative applied potential in this study was −0.55 V). We believe that the electrochemically stable Cu^+^/Cu^0^ interfaces across the reconstructed GBs were responsible for the low‐overpotential yet highly selective and productive CO_2_‐to‐C_2_H_4_ conversion over the CuO‐160W electrodes.

**Figure 5:**
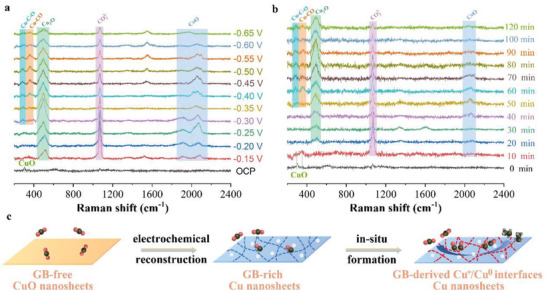
In situ Raman measurements of CuO‐160W electrode during eCO_2_RR. a) eCO_2_RR under different potentials for 10 min at each potential, b) eCO_2_RR under various durations at −0.50 V, and c) schematic illustration of the reconstruction mechanism of CuO‐160W under eCO_2_RR condition.

To further explore the surface reconstruction mechanism, we carried out in situ Raman spectroscopy to monitor the structural evolution of the CuO‐160W electrode. As shown in **Figure** **5**a, the typical Raman peaks of CuO at 250 and 280 cm^−1^ were observed for the CuO‐160W electrode at an open‐circuit potential (OCP). The CuO Raman signals disappeared when a constant potential of −0.15 V was applied. Meanwhile, a peak at 523 cm^−1^ was observed, which could be assigned to the Cu_2_O species.^[^ ^55^ ^]^ This Raman signal intensity of Cu_2_O continued to increase as the applied potential increased to −0.30 V and remained almost constant from −0.35 to −0.55 V. When the applied potential exceeded −0.55 V, the peak intensity of Cu_2_O slightly decreased but was still retained at a high potential of −0.65 V. The decrease in the peak intensity was ascribed to the applied potential exceeding the standard reduction potential of −0.36 V for bulk Cu_2_O reduction to Cu in an alkaline solution. The Raman spectra results indicated that the Cu_2_O species remained in existence during the eCO_2_RR, which was largely attributed to abundant undercoordinated Cu atoms or clusters in the dense Cu GBs.^[^ ^33^ ^]^ Further, we investigated time‐dependent Raman spectra on the CuO‐160W electrode at −0.50 V to gain in‐depth insight into the evolution of reconstruction with time (Figure 5b). The Cu_2_O Raman signal intensity increased as electrolysis extended, mainly because of the increased concentration of the reconstructed GBs with time. Combining these ex situ TEM and in situ Raman spectroscopy results, we propose that the CuO nanosheets are first reduced to metallic Cu. Thereafter, they are reconstructed to form Cu GBs, followed by reoxidization to Cu_2_O under eCO_2_RR conditions, affording electrochemically stable Cu^+^/Cu^0^ interfaces across the reconstructed Cu GBs regions (Figure 5e).

### 2.4 Mechanism of the CO_2_ Reduction at Low Overpotentials

**Figure 6:**
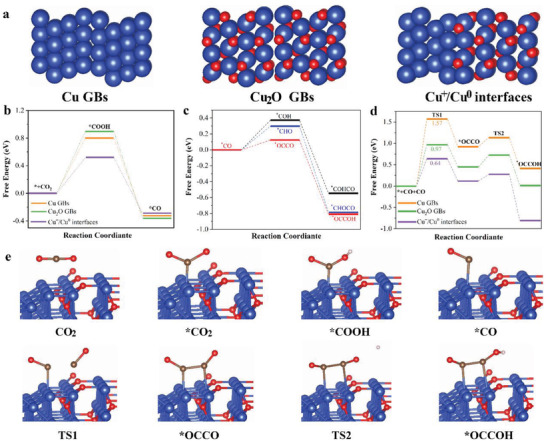
DFT calculations. a) Models for Cu GBs, Cu_2_O GBs, and Cu^+^/Cu^0^ interfaces. b) Free energy for hydrogenation of CO_2_ to form *CO on Cu GBs, Cu_2_O GBs, and Cu^+^/Cu^0^ interfaces. c) Free energy for the different pathways following *CO formation reduction, i.e., *CO reduction and *CO dimerization on Cu^+^/Cu^0^ interfaces. d) Energy diagram of *CO dimerization to *OCCO and the subsequent *OCCO hydrogenation to *OCCOH on Cu GBs, Cu_2_O GBs, and Cu^+^/Cu^0^ interfaces. e) Calculated optimized structures for main reaction intermediates on Cu^+^/Cu^0^ interfaces. The blue, gray, red, and white balls represent Cu, O, C, and H, respectively.

To gain mechanistic insight into the low overpotential CO_2_‐to‐C_2_H_4_ conversion on the Cu^+^/Cu^0^ interfaces, DFT calculations were performed to compare the thermodynamic reaction energies of the eCO_2_RR among the Cu GBs, Cu_2_O GBs, and Cu^+^/Cu^0^ interfaces (**Figure** **6**a). Cu(111) and Cu_2_O(111) were selected as the model planes based on the TEM results (Figure 4b). Since *CO is the key intermediate for the C—C coupling step, the Gibbs free energy of the CO_2_ reduction to *CO intermediates was first calculated for three structures of active sites: the Cu GBs, Cu_2_O GBs, and Cu^+^/Cu^0^ interfaces (Figure 6b). Along the pathway of the CO_2_ reduction to *CO, the Cu^+^/Cu^0^ interfaces required the lowest uphill reaction energy of 0.5 eV to form *COOH intermediates. The free‐energy diagram suggests that the Cu^+^/Cu^0^ interfaces facilitated the *CO formation more than the Cu GBs and Cu_2_O GBs. The following elemental reactions, involving the three branch steps of *CO dimerization, the hydrogenation of *CO to *COH, and the hydrogenation of *CO to *CHO intermediates, were further analyzed over the Cu GBs, Cu_2_O GBs, and Cu^+^/Cu^0^ interfaces (Figures S20–S22, Supporting Information). For the Cu GB surfaces, the reaction energy for the hydrogenation of *CO into *CHO was 0.44 eV (Figure S23, Supporting Information), which was lower than that for the dimerization of *CO to *OCCO (0.92 eV) and the hydrogenation of *CO into *COH (0.54 eV). This indicates that the C—C coupling step preferred the *CHO route over the *OCCO or *COH routes on the Cu GBs. The reaction energy for the *CO dimerization for the Cu_2_O GBs termination surface was reduced to 0.45 eV (Figure S24, Supporting Information), which was lower than that for the formation of *CHO (0.64 eV) and *COH (0.76 eV). Therefore, the dimerization of *CO to form *OCCO is an energetically favorable route for Cu_2_O GBs. Interestingly, the reaction energy of the *CO dimerization to *OCCO sharply declined to 0.12 eV on the Cu^+^/Cu^0^ interfaces, which was the lowest one compared to that for the formation of *COH and *CHO (Figure 6c). Therefore, the pathway of the dimerization of *CO to *OCCO was the most thermodynamically favorable for the Cu^+^/Cu^0^ interfaces. The stable Cu^+^/Cu^0^ interfaces were derived across the reconstructed Cu GBs, even under the eCO_2_RR.

We further studied the kinetics of the dimerization of *CO to *OCCO on the Cu GBs, Cu_2_O GBs, and Cu^+^/Cu^0^ interfaces, as shown in Figure 6d,e. The *CO dimerization on the Cu GBs surface required a high activation energy barrier (1.57 eV) to form a transient state (TS1). The activation energy barrier for TS1 decreased to 0.97 eV on the Cu_2_O GBs surfaces. It was further lowered to 0.64 eV on the Cu^+^/Cu^0^ interfaces, facilitating the dimerization of *CO to *OCCO. Additionally, the activation energy barrier for TS2 along the pathway of the hydrogenation of *OCCO to *OCCOH on the Cu^+^/Cu^0^ interfaces was lower than that for TS1, suggesting that *CO dimerization was the rate‐determining step (RDS) for eCO_2_RR. Further, previous studies found that the synergistic effect between the surface Cu^0^ and Cu^+^ sites promoted *CO dimerization to form *OCCO.^[^ ^10^ ^]^ The positively charged *CO on the Cu^+^ site and the negatively charged *CO on the Cu^0^ site were dimerized with feasible thermodynamics and kinetics.^[^ ^10^, ^22^ ^]^

Notably, the lowered activation energy barrier for the RDS of the *CO dimerization could not explain the low overpotential of CO_2_ reduction to C_2_H_4_ over the Cu^+^/Cu^0^ interfaces. The *CO dimerization rate had a reaction order of two with respect to the *CO surface coverage. Further, we compared the CO generation and dimerization rates as a function of the applied potential among three different CuO electrodes (Figure S25, Supporting Information). The CuO‐160W electrode containing the Cu^+^/Cu^0^ interfaces showed the highest CO generation rate of 83 mA cm^−2^ at a low potential of −0.52 V, translating to the highest *CO surface coverage. The in situ Raman spectra results supported the high *CO surface coverage of the CuO‐160W electrode. Upon applying a potential ranging from −0.30 to −0.65 V, three new bands at 280, 365, and 1900–2200 cm^−1^ were observed, which were assigned to the CO—Cu frustrated rotation, CO—Cu stretch, and C≡O stretch, respectively (Figure 5a).^[^ ^56^ ^]^ The integrated area of the C≡O stretching band was directly proportional to the *CO surface coverage.^[^ ^57^ ^]^ The CuO‐160W electrode exhibited higher *CO surface coverage than the CuO‐0W and CuO‐80W electrodes according to the integrated area of the C≡O stretch band (Figure 5a; Figure S26, Supporting Information). Resultantly, the *CO dimerization rate was not limited by accessible *CO, affording a low overpotential for CO_2_ to C_2_H_4_ reduction over the CuO‐160W electrode. Notably, the relatively high *CO surface coverage further boosted the *CO dimerization rate for the CuO‐160W electrode.^[^ ^58^, ^59^ ^]^ The *CO dimerization rate of the CuO‐160W electrode was 3.7 times higher than that of the CuO‐0W electrode at −0.52 V (Figure S25, Supporting Information).

## 3 Conclusion

In summary, CuO nanosheets synthesized by the ultrasonication‐assisted electrodeposition method are susceptible to in situ restructuring to form high‐density GBs with Cu^+^/Cu^0^ interfaces during the eCO_2_RR. Combining experimental results with DFT calculations, we found that the Cu^+^/Cu^0^ interfaces across the region of reconstructed GBs reduced the kinetic energy barrier of the C—C coupling through the *CO dimerization. The unique structure of the reconstructed GBs with the Cu^+^/Cu^0^ interfaces provided abundant active sites for the CO_2_ activation to produce more CO intermediates in the low overpotential region. The high concentration of the CO intermediates translated to a high *CO surface coverage, further reducing the activation energy barrier of the C—C coupling. Overall, high *CO surface coverage circumvented the limitation of accessible *CO and allowed the *CO dimerization at low overpotentials. Resultantly, the CuO nanosheet electrode achieved a remarkable $FE_{C_{2}H_{4}}$ of 62.5% with a current density of 173 mA cm^−2^ at a relatively low potential of −0.52 V. Such a low overpotential and high $FE_{C_{2}H_{4}}$ yielded a high half‐cell CEE of 41% for C_2_H_4_ formation.

## 4 Experimental Section

### Catalyst Synthesis

The CuO catalysts were synthesized using an ultrasonic‐assisted electrodeposition method. The electrodeposition was conducted in a two‐electrode cell equipped with a Cu‐foam anode and carbon‐paper cathode. Further, 3.0 m KOH solution was used as the electrolyte. The galvanostatic mode was employed for the electrodeposition of Cu samples on the carbon paper. The accumulated Cu deposits were exfoliated from the carbon paper under an ultrasonic process at 160 W and spontaneously oxidized into CuO in the KOH solution. Five CuO‐160W catalysts were synthesized with electrodeposition current densities of 160, 180, 190, 200, and 220 mA cm^−2^. Two control CuO samples were prepared with ultrasonic powers of 0 and 80 W under an electrodeposition current density of 190 mA cm^−2^.

### Physical Characterization

SEM images were collected using an SU8020 microscope to analyze the morphology of the catalysts. STEM was performed using an FEI Talos 200F microscope with an acceleration voltage of 200 kV and an HAADF detector. XRD was performed using a PANalytical X‐Pert PRO MPD instrument with a Cu‐target X‐ray source (*λ* = 1.5 Å). XPS was performed on an ESCAL AB250Xi spectrometer with Al K*α* X‐ray radiation. X‐ray absorption spectroscopy measurements at the Cu K‐edge were conducted at the National Synchrotron Light Source II of the Brookhaven National Laboratory. In situ Raman spectra were collected on a Horiba LabRAM HR evolution Raman spectrometer with a 633 nm laser equipped with a gas–solid–liquid three‐phase flow cell (Gaossunion Technology Co., Ltd.). During the in situ Raman testing, a 1.0 m KOH solution serving as both the catholyte and anolyte was pumped into the electrolyte compartment at a flow rate of 0.1 mL min^−1^, whereas CO_2_ (99.99%) was continuously supplied to the gas compartment at a flow rate of 5 sccm.

### ECO_2_RR Measurements

The eCO_2_RR experiments were performed on a customized electrochemical test station equipped with a Gamry electrochemical workstation, homemade flow cell electrolyzer, mass flow controller, and peristaltic pump. CuO catalyst inks were sprayed onto a 2 × 2 cm^2^ gas diffusion layer (GDL), a carbon paper with a microporous carbon layer (Sigracet 34BC), to prepare gas diffusion electrodes (GDEs). The loading of the CuO catalysts was 0.5 mg cm^−2^ for all GDEs. The as‐prepared, relatively large CuO‐based GDEs were cut into 1 × 1 cm^2^ pieces, which served as the cathode for multiple independent tests, whereas Ni foam pressed onto a 2 × 2 cm^2^ GDL acted as the anode. The current densities were calculated based on the active geometric area of the cathode (1 cm^2^). Additionally, 1.0 m KOH solution was pumped into the cathodic and anodic compartments at a flow rate of 1.0 mL min^−1^ and separated using an anion exchange membrane (Fumasep FAA‐3‐PK‐75). High‐purity CO_2_ gas was fed into the serpentine flow channel on the cathode side at a flow rate of 50.0 sccm. A constant cell voltage was applied to the flow cell during CO_2_ electrolysis. An Ag/AgCl (saturated KCl) electrode was bridged to the cathodic compartment to measure the electrode potential. All potentials were converted to an RHE scale with manual *iR*_s_ compensation: *E*_RHE_ = *E*_Ag/AgCl_ + 0.0591 × pH + 0.197 V − *iR*_s_. The *iR*_s_ was determined by potentiostatic electrochemical impedance spectroscopy measurements under an OCP at frequencies ranging from 100 kHz to 0.1 Hz.

### Product Analysis

The gaseous products were analyzed by online gas chromatography (GC, Agilent 7890 B) with a flame ionization detector (FID) and a thermal conductivity detector (TCD). The molar percentages of the gaseous products were calculated from the GC peak areas based on the calibration curves (TCD for H_2_, CO_2_, and CO; FID for CH_4_, C_2_H_4_, and C_2_H_6_). The liquid products were quantified by ^1^H nuclear magnetic resonance (NMR) spectroscopy (Bruker AV 400 MHz spectrometer). The concentration of the liquid products was calculated based on the NMR peak integral areas and calibration curves. To prepare the NMR samples, 500 µL of the collected electrolyte was mixed with 100 µL of D_2_O solution consisting of 5 × 10^−3^ m 3‐(trimethylsilyl)propionic‐2,2,3,3‐d_4_ acid sodium salt (TSP). TSP was used as an internal standard.

The half‐cell cathodic energy efficiency was calculated by the following equation

(1) $$\mathrm{CEE}=\frac{\left(1.23-E_{C_{2}H_{4}}^{0}\right)\times FE_{C_{2}H_{4}}}{1.23-E_{\mathrm{cathode}}}$$

where the overpotential of anodic OER is assumed to be zero, $E_{C_{2}H_{4}}^{0}$denotes the standard potential of CO_2_ reduction to C_2_H_4_, 0.08 V, $FE_{C_{2}H_{4}}$ is the FE of C_2_H_4_, and *E*_cathode_ is the applied potential of the cathode.

### DFT Calculations

First‐principles were employed to perform all spin‐polarization DFT calculations within the generalized gradient approximation using the Perdew–Burke–Ernzerhof formulation.^[^ ^60^, ^61^ ^]^ The projected augmented wave potentials were chosen to describe the ionic cores and considered valence electrons using a plane‐wave basis set with a kinetic energy cutoff of 400 eV.^[^ ^62^ ^]^ The symmetrical low‐angle tilt boundary model was chosen for the DFT simulation based on the HRTEM images. The partial occupancies of the Kohn–Sham orbitals were allowed using the Gaussian smearing method with a width of 0.05 eV. The electronic energy was considered to be self‐consistent when the energy change was less than 10^−4 ^eV. Geometry optimization was deemed to be convergent when the energy change was smaller than 0.04 eV Å^−1^. The vacuum spacing in the direction perpendicular to the plane of the structure was 16 Å. Brillouin zone integration was performed using 2 × 2 × 1 Monkhorst–Pack *k*‐point sampling for the structure. Gibbs free energy was calculated using the following equation

(2) G=E+ZPE−TS

where *G*, *E*, ZPE, and TS are the Gibbs free energy, total energy from DFT calculations, zero‐point energy, and entropic contributions, respectively. The U correction was set to 3.41 eV for the Cu atoms in the systems.

## Conflict of Interest

The authors declare no conflict of interest.

## Supporting information

Supporting Information

## Data Availability

The data that support the findings of this study are available from the corresponding author upon reasonable request.

## References

[1] M. Liu , Y. Pang , B. Zhang , P. De Luna , O. Voznyy , J. Xu , X. Zheng , C. T. Dinh , F. Fan , C. Cao , F. P. de Arquer , T. S. Safaei , A. Mepham , A. Klinkova , E. Kumacheva , T. Filleter , D. Sinton , S. O. Kelley , E. H. Sargent , Nature 2016, 537, 382. 27487220 10.1038/nature19060

[2] H. Mistry , A. S. Varela , S. Kühl , P. Strasser , B. R. Cuenya , Nat. Rev. Mater. 2016, 1, 16009.

[3] H. Shin , K. U. Hansen , F. Jiao , Nat. Sustainability 2021, 4, 911.

[4] F. Li , A. Thevenon , A. Rosas‐Hernandez , Z. Wang , Y. Li , C. M. Gabardo , A. Ozden , C. T. Dinh , J. Li , Y. Wang , J. P. Edwards , Y. Xu , C. McCallum , L. Tao , Z. Q. Liang , M. Luo , X. Wang , H. Li , C. P. O'Brien , C. S. Tan , D. H. Nam , R. Quintero‐Bermudez , T. T. Zhuang , Y. C. Li , Z. Han , R. D. Britt , D. Sinton , T. Agapie , J. C. Peters , E. H. Sargent , Nature 2020, 577, 509. 31747679 10.1038/s41586-019-1782-2

[5] M. Zhong , K. Tran , Y. Min , C. Wang , Z. Wang , C. T. Dinh , P. De Luna , Z. Yu , A. S. Rasouli , P. Brodersen , S. Sun , O. Voznyy , C. S. Tan , M. Askerka , F. Che , M. Liu , A. Seifitokaldani , Y. Pang , S. C. Lo , A. Ip , Z. Ulissi , E. H. Sargent , Nature 2020, 581, 178. 32405017 10.1038/s41586-020-2242-8

[6] X. Chen , J. Chen , N. M. Alghoraibi , D. A. Henckel , R. Zhang , U. O. Nwabara , K. E. Madsen , P. J. A. Kenis , S. C. Zimmerman , A. A. Gewirth , Nat. Catal. 2020, 4, 20.

[7] C. W. Li , M. W. Kanan , J. Am. Chem. Soc. 2012, 134, 7231. 22506621 10.1021/ja3010978

[8] K. Zhao , Y. Liu , X. Quan , S. Chen , H. Yu , ACS Appl. Mater. Interfaces 2017, 9, 5302. 28103017 10.1021/acsami.6b15402

[9] T. C. Chou , C. C. Chang , H. L. Yu , W. Y. Yu , C. L. Dong , J. J. Velasco‐Velez , C. H. Chuang , L. C. Chen , J. F. Lee , J. M. Chen , H. L. Wu , J. Am. Chem. Soc. 2020, 142, 2857. 31955572 10.1021/jacs.9b11126

[10] H. Xiao , W. A. Goddard, III , T. Cheng , Y. Liu , Proc. Natl. Acad. Sci. USA 2017, 114, 6685. 28607069 10.1073/pnas.1702405114 PMC5495255

[11] H. Mistry , A. S. Varela , C. S. Bonifacio , I. Zegkinoglou , I. Sinev , Y. W. Choi , K. Kisslinger , E. A. Stach , J. C. Yang , P. Strasser , B. R. Cuenya , Nat. Commun. 2016, 7, 12123. 27356485 10.1038/ncomms12123 PMC4931497

[12] Z. Chen , T. Wang , B. Liu , D. Cheng , C. Hu , G. Zhang , W. Zhu , H. Wang , Z. J. Zhao , J. Gong , J. Am. Chem. Soc. 2020, 142, 6878. 32220209 10.1021/jacs.0c00971

[13] X. Feng , K. Jiang , S. Fan , M. W. Kanan , J. Am. Chem. Soc. 2015, 137, 4606. 25835085 10.1021/ja5130513

[14] B. Zhang , J. Zhang , M. Hua , Q. Wan , Z. Su , X. Tan , L. Liu , F. Zhang , G. Chen , D. Tan , X. Cheng , B. Han , L. Zheng , G. Mo , J. Am. Chem. Soc. 2020, 142, 13606. 32658474 10.1021/jacs.0c06420

[15] Z. Gu , H. Shen , Z. Chen , Y. Yang , C. Yang , Y. Ji , Y. Wang , C. Zhu , J. Liu , J. Li , T.‐K. Sham , X. Xu , G. Zheng , Joule 2021, 5, 429.

[16] R. M. Arán‐Ais , F. Scholten , S. Kunze , R. Rizo , B. Roldan Cuenya , Nat. Energy 2020, 5, 317.

[17] C. Choi , T. Cheng , M. Flores Espinosa , H. Fei , X. Duan , W. A. Goddard, III , Y. Huang , Adv. Mater. 2019, 31, 1805405. 10.1002/adma.201805405 30549121

[18] K. K. Patra , S. Park , H. Song , B. Kim , W. Kim , J. Oh , ACS Appl. Energy Mater. 2020, 3, 11343.

[19] A. Chen , X. Yu , Y. Zhou , S. Miao , Y. Li , S. Kuld , J. Sehested , J. Liu , T. Aoki , S. Hong , M. F. Camellone , S. Fabris , J. Ning , C. Jin , C. Yang , A. Nefedov , C. Wöll , Y. Wang , W. Shen , Nat. Catal. 2019, 2, 334.

[20] H. Li , T. Liu , P. Wei , L. Lin , D. Gao , G. Wang , X. Bao , Angew. Chem., Int. Ed. 2021, 60, 14329. 10.1002/anie.202102657 33837619

[21] C. Kim , K. M. Cho , K. Park , J. Y. Kim , G. T. Yun , F. M. Toma , I. Gereige , H. T. Jung , Adv. Funct. Mater. 2021, 31, 2102142.

[22] J. Jiao , R. Lin , S. Liu , W. C. Cheong , C. Zhang , Z. Chen , Y. Pan , J. Tang , K. Wu , S. F. Hung , H. M. Chen , L. Zheng , Q. Lu , X. Yang , B. Xu , H. Xiao , J. Li , D. Wang , Q. Peng , C. Chen , Y. Li , Nat. Chem. 2019, 11, 222. 30664719 10.1038/s41557-018-0201-x

[23] S. H. Lee , J. C. Lin , M. Farmand , A. T. Landers , J. T. Feaster , J. E. Aviles Acosta , J. W. Beeman , Y. Ye , J. Yano , A. Mehta , R. C. Davis , T. F. Jaramillo , C. Hahn , W. S. Drisdell , J. Am. Chem. Soc. 2021, 143, 588. 33382947 10.1021/jacs.0c10017

[24] A. Eilert , F. Cavalca , F. S. Roberts , J. Osterwalder , C. Liu , M. Favaro , E. J. Crumlin , H. Ogasawara , D. Friebel , L. G. Pettersson , A. Nilsson , J. Phys. Chem. Lett. 2017, 8, 285. 27983864 10.1021/acs.jpclett.6b02273

[25] Y. Lum , J. W. Ager , Angew. Chem., Int. Ed. 2018, 57, 551. 10.1002/anie.201710590 29110417

[26] S. C. Lin , C. C. Chang , S. Y. Chiu , H. T. Pai , T. Y. Liao , C. S. Hsu , W. H. Chiang , M. K. Tsai , H. M. Chen , Nat. Commun. 2020, 11, 3525. 32665607 10.1038/s41467-020-17231-3 PMC7360608

[27] A. J. Garza , A. T. Bell , M. Head‐Gordon , J. Phys. Chem. Lett. 2018, 9, 601. 29341623 10.1021/acs.jpclett.7b03180

[28] M. Fields , X. Hong , J. K. Nørskov , K. Chan , J. Phys. Chem. C 2018, 122, 16209.

[29] F. Cavalca , R. Ferragut , S. Aghion , A. Eilert , O. Diaz‐Morales , C. Liu , A. L. Koh , T. W. Hansen , L. G. M. Pettersson , A. Nilsson , J. Phys. Chem. C 2017, 121, 25003.

[30] C. Liu , S. Hedström , J. H. Stenlid , L. G. M. Pettersson , J. Phys. Chem. C 2019, 123, 4961.

[31] G. Liu , M. Lee , S. Kwon , G. Zeng , J. Eichhorn , A. K. Buckley , F. D. Toste , W. A. Goddard, III , F. M. Toma , Proc. Natl. Acad. Sci. USA 2021, 118, e2012649118. 34083432 10.1073/pnas.2012649118 PMC8201769

[32] F. Dattila , R. García‐Muelas , N. López , ACS Energy Lett. 2020, 5, 3176.

[33] B. G. Ershov , E. Janata , M. Michaelis , A. Henglein , J. Phys. Chem. 1991, 95, 8996.

[34] H. Jung , S. Y. Lee , C. W. Lee , M. K. Cho , D. H. Won , C. Kim , H. S. Oh , B. K. Min , Y. J. Hwang , J. Am. Chem. Soc. 2019, 141, 4624. 30702874 10.1021/jacs.8b11237

[35] Z. Xu , T. Wu , Y. Cao , C. Chen , X. Zeng , P. Lin , W.‐W. Zhao , J. Catal. 2020, 383, 42.

[36] Q. Lei , H. Zhu , K. Song , N. Wei , L. Liu , D. Zhang , J. Yin , X. Dong , K. Yao , N. Wang , X. Li , B. Davaasuren , J. Wang , Y. Han , J. Am. Chem. Soc. 2020, 142, 4213. 32041401 10.1021/jacs.9b11790

[37] W. T. Osowiecki , J. J. Nussbaum , G. A. Kamat , G. Katsoukis , M. Ledendecker , H. Frei , A. T. Bell , A. P. Alivisatos , ACS Appl. Energy Mater. 2019, 2, 7744.

[38] J. Huang , N. Hormann , E. Oveisi , A. Loiudice , G. L. De Gregorio , O. Andreussi , N. Marzari , R. Buonsanti , Nat. Commun. 2018, 9, 3117. 30082872 10.1038/s41467-018-05544-3 PMC6079067

[39] H. Magnusson , K. Frisk , *Self‐Diffusion and Impurity Diffusion of Hydrogen, Oxygen, Sulphur and Phosphorus in Copper*, Technical Report, SKB TR‐13‐24, Stockholm, Swedish 2013.

[40] I. Tudela , Y. Zhang , M. Pal , I. Kerr , A. J. Cobley , Surf. Coat. Tech. 2014, 259, 363.

[41] Y. Wang , H. Shen , K. J. T. Livi , D. Raciti , H. Zong , J. Gregg , M. Onadeko , Y. Wan , A. Watson , C. Wang , Nano Lett. 2019, 19, 8461. 31671267 10.1021/acs.nanolett.9b02748

[42] R.‐X. Yang , Y.‐R. Wang , G.‐K. Gao , L. Chen , Y. Chen , S.‐L. Li , Y.‐Q. Lan , Small Struct. 2021, 2, 2100012.

[43] T. T. H. Hoang , S. Verma , S. Ma , T. T. Fister , J. Timoshenko , A. I. Frenkel , P. J. A. Kenis , A. A. Gewirth , J. Am. Chem. Soc. 2018, 140, 5791. 29620896 10.1021/jacs.8b01868

[44] G. L. De Gregorio , T. Burdyny , A. Loiudice , P. Iyengar , W. A. Smith , R. Buonsanti , ACS Catal. 2020, 10, 4854. 32391186 10.1021/acscatal.0c00297 PMC7199425

[45] S. Ma , M. Sadakiyo , R. Luo , M. Heima , M. Yamauchi , P. J. A. Kenis , J. Power Sources 2016, 301, 219.

[46] G. Zhou , L. Wang , J. C. Yang , J. Appl. Phys. 2005, 97, 063509.

[47] K. Lahtonen , M. Hirsimaki , M. Lampimaki , M. Valden , J. Chem. Phys. 2008, 129, 124703. 19045044 10.1063/1.2980347

[48] J. Nowotny , W. Weppner , Non‐Stoichiometric Compounds Surfaces, Grain Boundaries and Structural Defects, NATO ASI Series, Kluwer Academic Publishers, Dordrecht, The Netherlands 1989.

[49] A. Henglein , Phys. Chem. 1990, 94, 5169.

[50] A. Fojtik , A. Henglein , E. Janata , J. Phys. Chem. 1992, 96, 8203.

[51] P. Jaque , A. V. Marenich , C. J. Cramer , D. G. Truhlar , J. Phys. Chem. C 2007, 111, 5783.

[52] W. J. Plieth , J. Phys. Chem. 1982, 86, 3166.

[53] S. Mosseri , J. Phys. Chem. 1989, 93, 6791.

[54] A. Henglein , Ber. Bunsenges. Phys. Chem. 1977, 81, 556.

[55] H. S. Jeon , J. Timoshenko , C. Rettenmaier , A. Herzog , A. Yoon , S. W. Chee , S. Oener , U. Hejral , F. T. Haase , B. Roldan Cuenya , J. Am. Chem. Soc. 2021, 143, 7578. 33956433 10.1021/jacs.1c03443 PMC8154520

[56] C. Chen , X. Yan , Y. Wu , S. Liu , X. Sun , Q. Zhu , R. Feng , T. Wu , Q. Qian , H. Liu , L. Zheng , J. Zhang , B. Han , Chem. Sci. 2021, 12, 5938. 35342541 10.1039/d1sc00042j PMC8869928

[57] C. M. Gunathunge , X. Li , J. Li , R. P. Hicks , V. J. Ovalle , M. M. Waegele , J. Phys. Chem. C 2017, 121, 12337.

[58] R. B. Sandberg , J. H. Montoya , K. Chan , J. K. Nørskov , Surf. Sci. 2016, 654, 56.

[59] F. Li , Y. C. Li , Z. Wang , J. Li , D.‐H. Nam , Y. Lum , M. Luo , X. Wang , A. Ozden , S.‐F. Hung , B. Chen , Y. Wang , J. Wicks , Y. Xu , Y. Li , C. M. Gabardo , C.‐T. Dinh , Y. Wang , T.‐T. Zhuang , D. Sinton , E. H. Sargent , Nat. Catal. 2019, 3, 75.

[60] G. Kresse , J. Furthmuller , Comput. Mater. Sci. 1996, 6, 15.

[61] G. Kresse , J. Furthmuller , Phys. Rev. B 1996, 54, 11169. 10.1103/physrevb.54.11169 9984901

[62] G. Kresse , D. Joubert , Phys. Rev. B 1999, 59, 1758.

