# Supplementary material for: Grain Boundary‐Derived Cu+/Cu0 Interfaces in CuO Nanosheets for Low Overpotential Carbon Dioxide Electroreduction to Ethylene
Source: Adv Sci (Weinh). 2022 May 22;9(21):2200454. doi: 10.1002/advs.202200454 (PMC9313501; doi:10.1002/advs.202200454)
Supplement: Supplementary file 1 — Supporting Information [file ADVS-9-2200454-s001.pdf]

ADVANCED SCIENCE
Open Access

Supporting Information

for *Adv. Sci.*, DOI 10.1002/advs.202200454

Grain Boundary-Derived Cu^{+}/Cu^{0} Interfaces in CuO Nanosheets for Low Overpotential Carbon Dioxide Electroreduction to Ethylene

Jianfang Zhang, Yan Wang, Zhengyuan Li, Shuai Xia, Rui Cai, Lu Ma, Tianyu Zhang, Josh Ackley, Shize Yang*, Yucheng Wu* and Jingjie Wu**

Supporting Information

Grain boundary-derived Cu^{+}/Cu^{0} interfaces in CuO nanosheets for low overpotential CO_2 electroreduction to ethylene

Jianfang Zhang, Yan Wang, Zhengyuan Li, Shuai Xia, Rui Cai, Lu Ma, Tianyu Zhang, Josh Ackley, Shize Yang*, Yucheng Wu*, Jingjie Wu**

Dr. J. F. Zhang, S. Xia, R. Cai, Dr. Y. Wang, Prof. Y. C. Wu

School of Materials Science and Engineering, Hefei University of Technology, Hefei 230009, China

E-mail: stone@hfut.edu.cn

Prof. Y. C. Wu

China International S&T Cooperation Base for Advanced Energy and Environmental Materials & Anhui Provincial International S&T Cooperation Base for Advanced Energy Materials, Hefei University of Technology, Hefei 230009, China.

E-mail: ycwu@hfut.edu.cn

Dr. J. F. Zhang, Z. Y. Li, T. Y. Zhang, J. Ackley, Prof. J. J. Wu

Department of Chemical and Environmental Engineering, University of Cincinnati, Cincinnati, OH 45221, United States

E-mail: jingjie.wu@uc.edu

Dr. S. Z. Yang

Eyring Materials Center, Arizona State University, Tempe, Arizona 85287, United States

E-mail: shize.yang@asu.edu

Dr. L. Ma

National Synchrotron Light Source II, Brookhaven National Laboratory, Upton, New York, 11971, United States

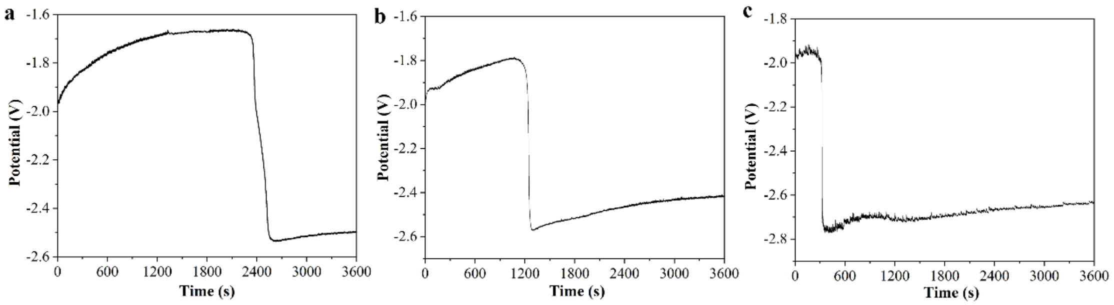

Figure S1. The chronopotentiometric E-t curves of the synthesis process for CuO nanosheets at different ultrasonic power. (a) 160 W, (b) 80 W, and (c) 0 W.

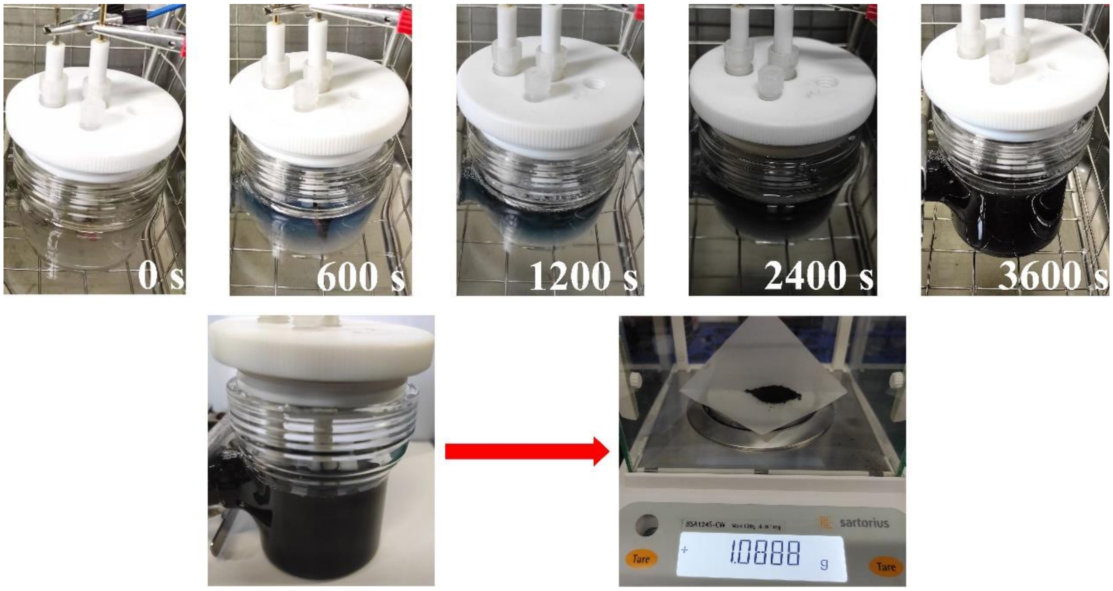

Figure S2. Photo images of the color change of KOH electrolyte with the electrodeposition time during the synthesis of CuO-160W nanosheets.

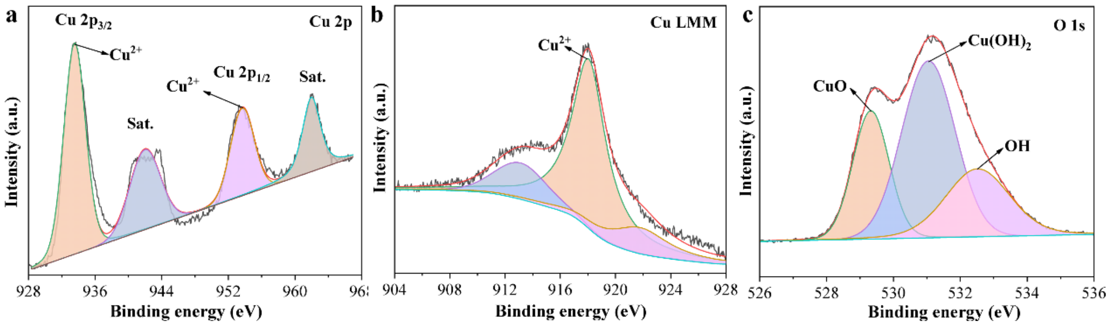

Figure S3. XPS spectra of as-prepared CuO-160W nanosheets. (a) Cu 2p, (b) Cu LMM, and (c) O1s.

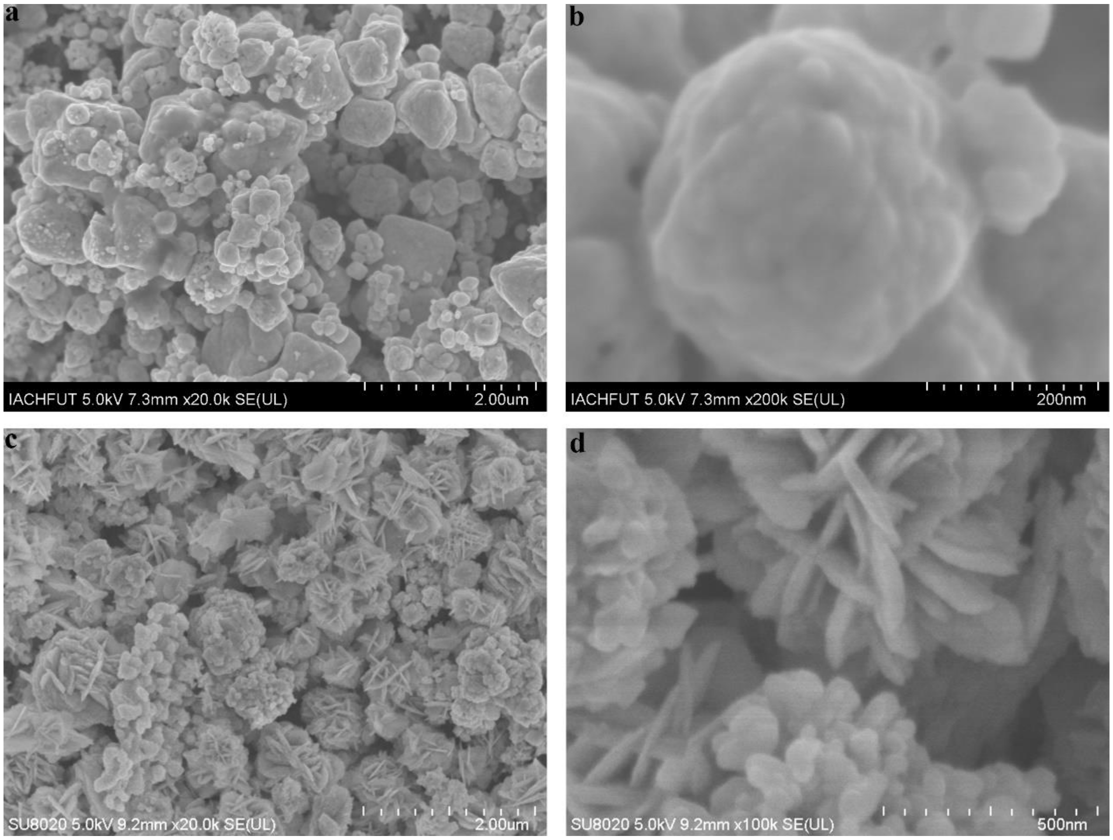

Figure S4. SEM images of as-prepared CuO catalysts at different ultrasonic power. (a-b) CuO-0W, and (c-d) CuO-80W.

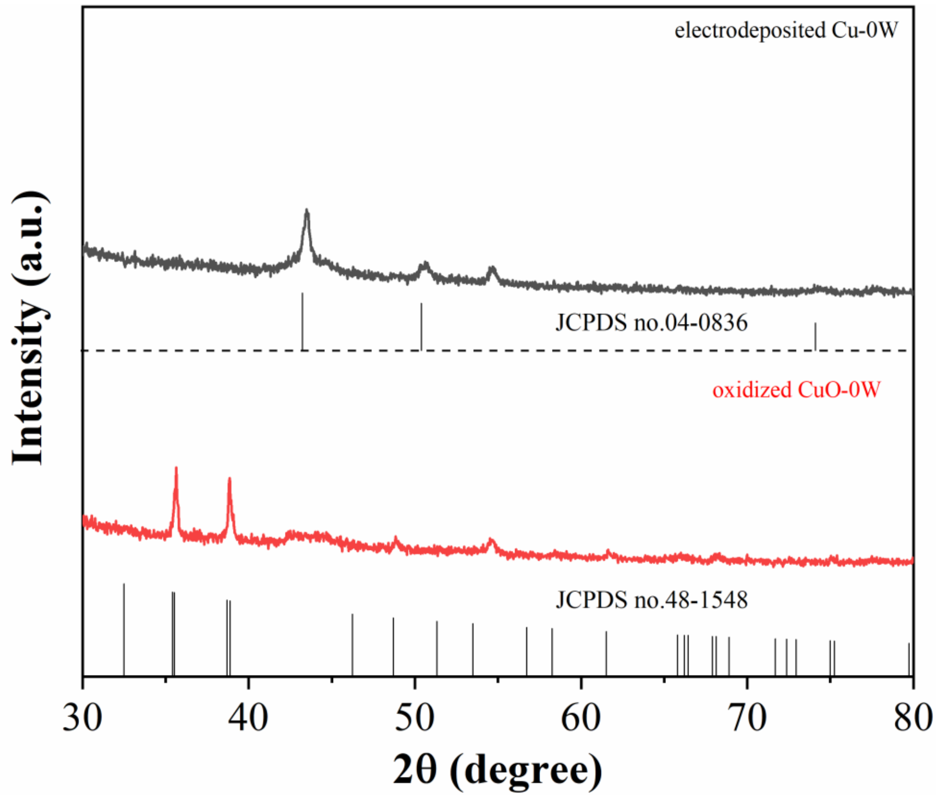

Figure S5. XRD patterns of the electrodeposited Cu-0W before and after spontaneous oxidation in KOH solution.

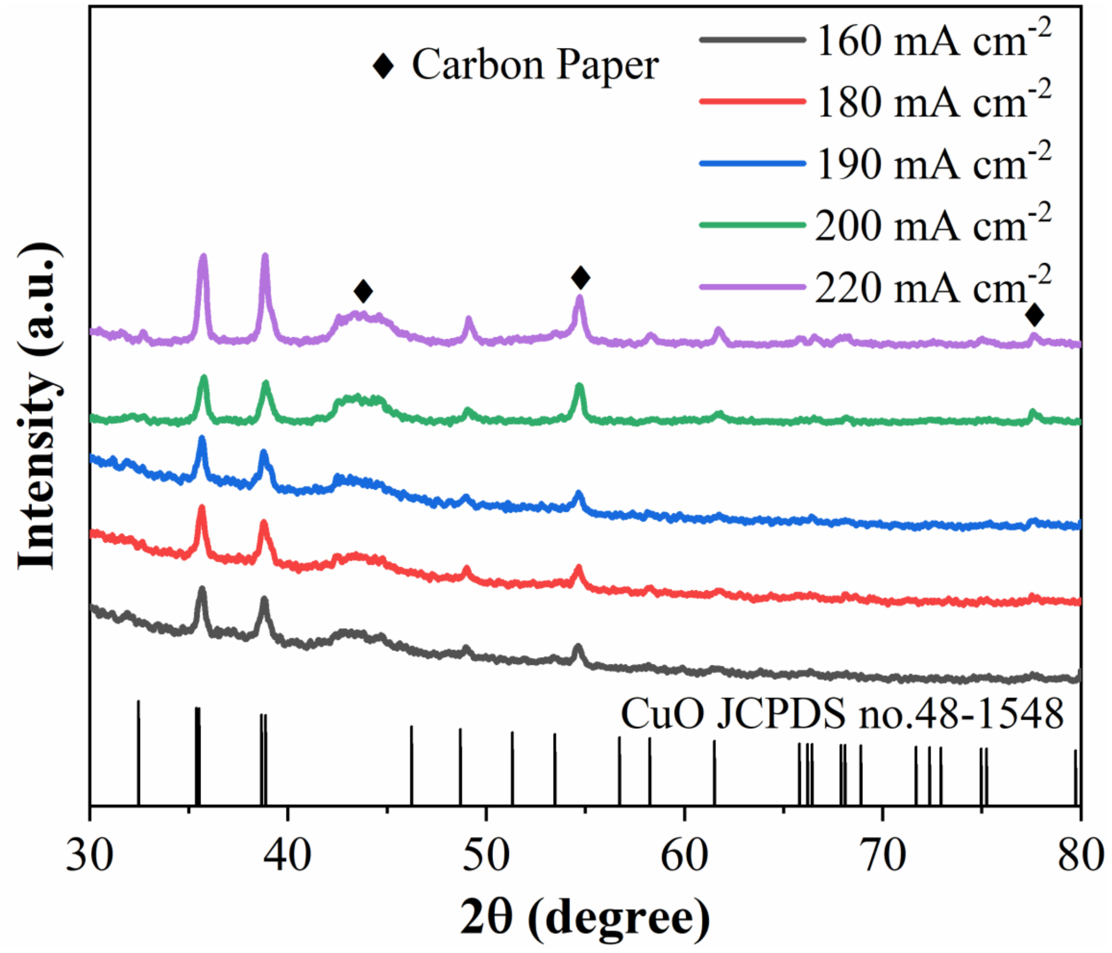

Figure S6. XRD patterns of various CuO-160W catalysts prepared under different electrodeposition current densities and a fixed ultrasonic power of 160 W.

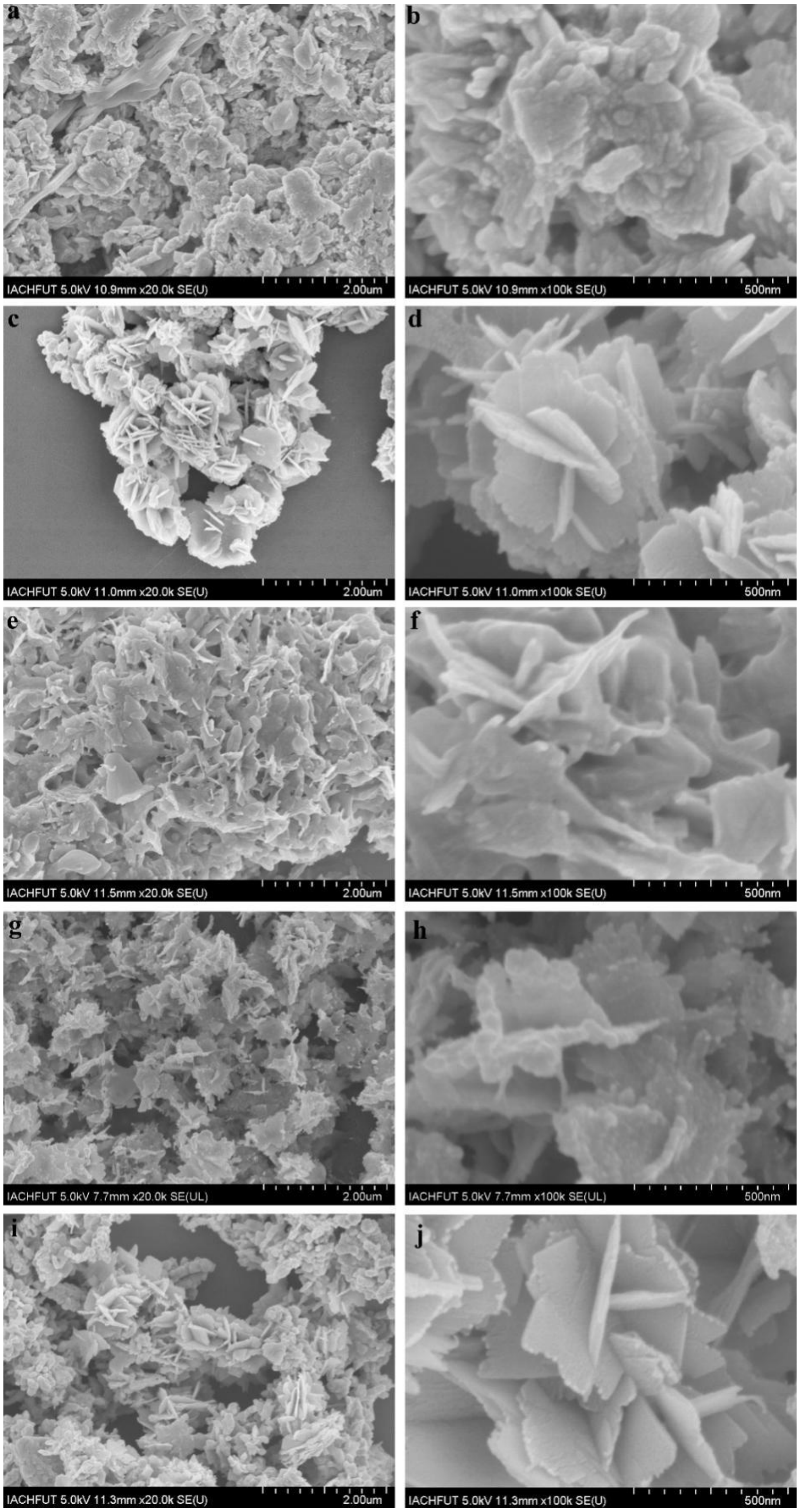

Figure S7. SEM images of various CuO-160W catalysts prepared by under different electrodeposition current densities and a fixed ultrasonic power of 160 W. (a,b) 160 mA cm^{-2}, (c,d) 180 mA cm^{-2}, (e,f) 190 mA cm^{-2}, (g,h) 200 mA cm^{-2}, and (i, j) 220 mA cm^{-2}.

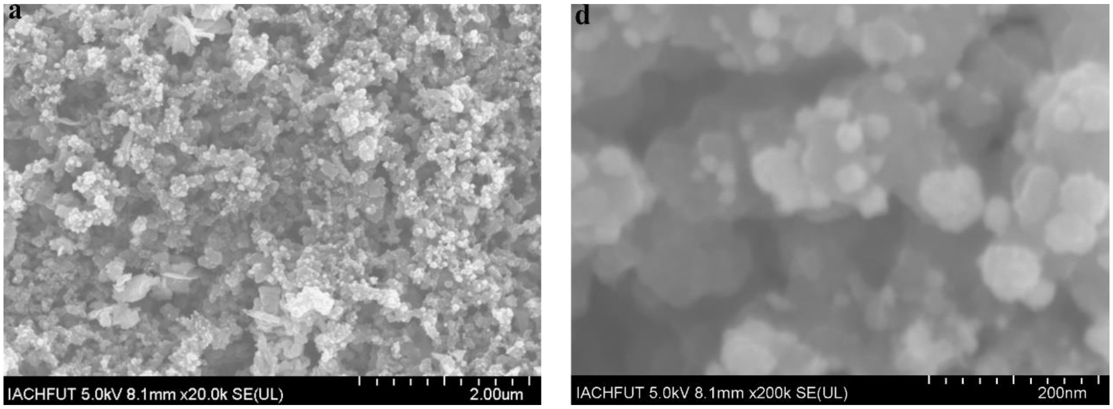

Figure S8. SEM images of the electrodeposited Cu on the carbon paper at 190 mA cm^{-2} and 160 W.

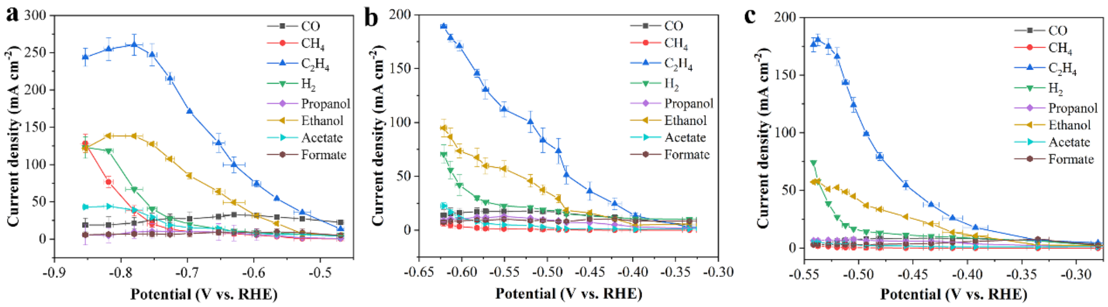

Figure S9. The partial current densities of CO_2 reduction products on (a) CuO-0W, (b) CuO-80W, and (c) CuO-160W electrodes.

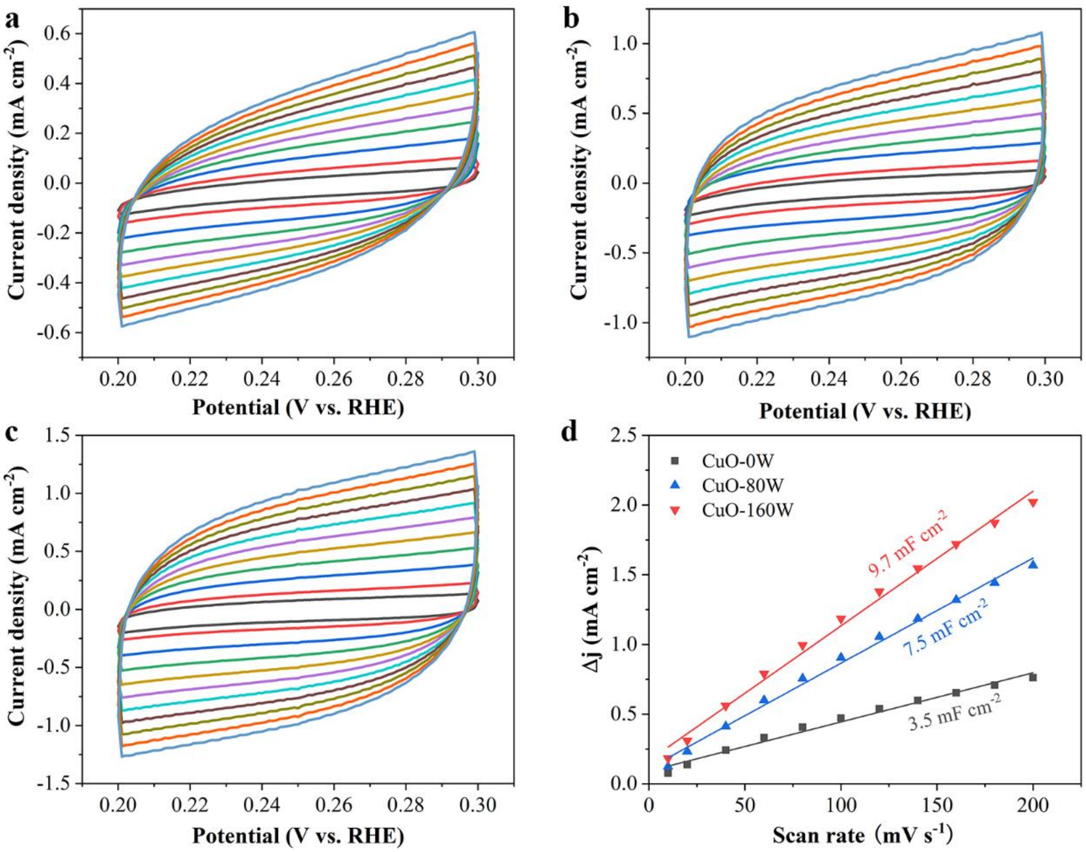

Figure S10. Cyclic voltametry (CV) curves of different CuO electrodes at various scan rates from 10 to 200 mV s^{-1}. (a) CuO-0W, (b) CuO-80W, and (c) CuO-160W, (d) the corresponding C_{dl} calculated from CV curves.

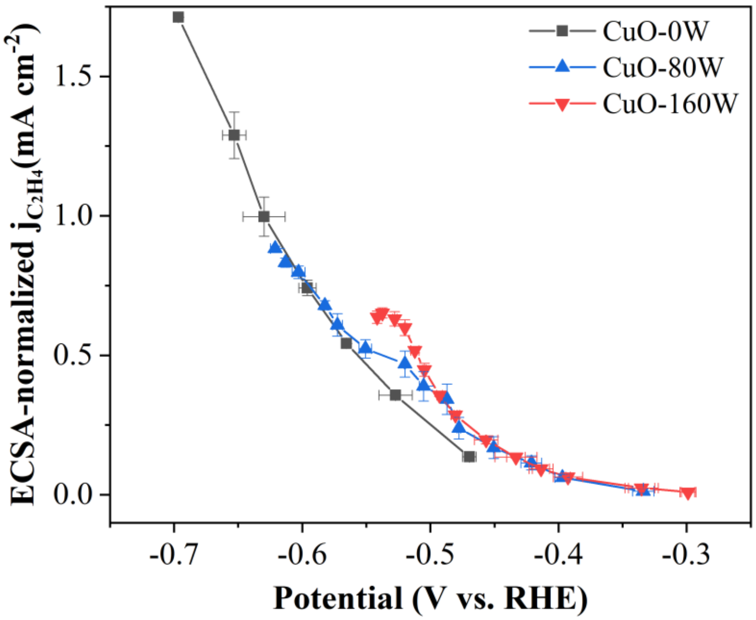

Figure S11. ECSA-normalized j_{C2H4} of CuO-0W, CuO-80W, and CuO-160W electrodes.

(Note: ECSA=Geometric area (cm^2) * RF; where RF is the roughness factor, RF= C_{dl}/C_f, C_f is 35 μF cm^{-2} for Cu foil)

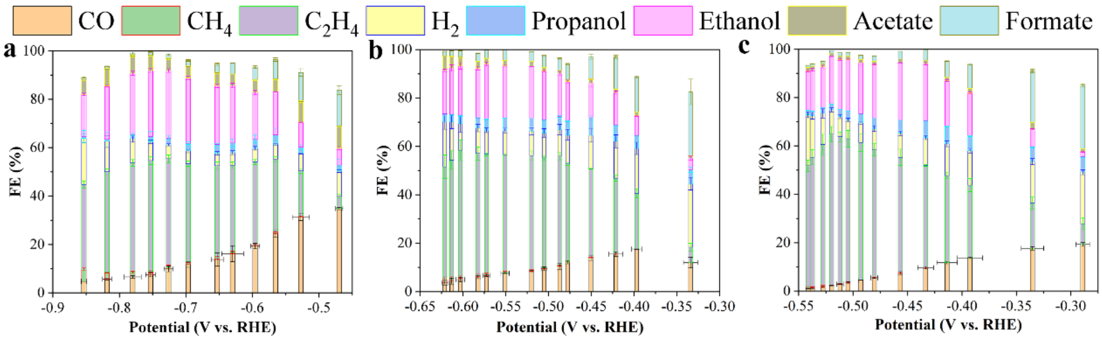

Figure S12. The faradaic efficiencies of CO_2 reduction products on (a) CuO-0W, (b) CuO-80W, and (c) CuO-160W electrodes.

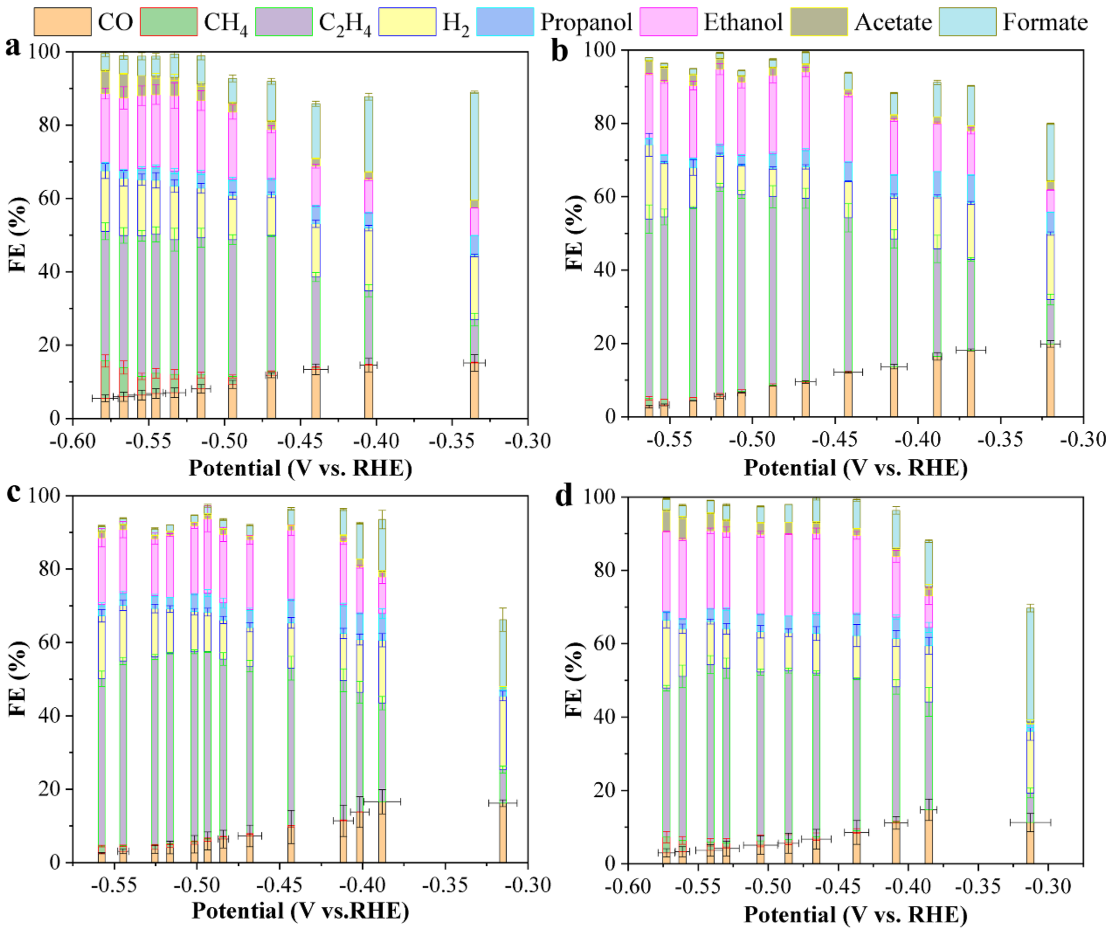

Figure S13. The faradaic efficiencies of CO_2 reduction products on CuO-160W electrodes prepared at different electrodeposition current densities. (a) 160 mA cm^{-2}, (b) 180 mA cm^{-2}, (c) 200 mA cm^{-2}, and (d) 220 mA cm^{-2}.

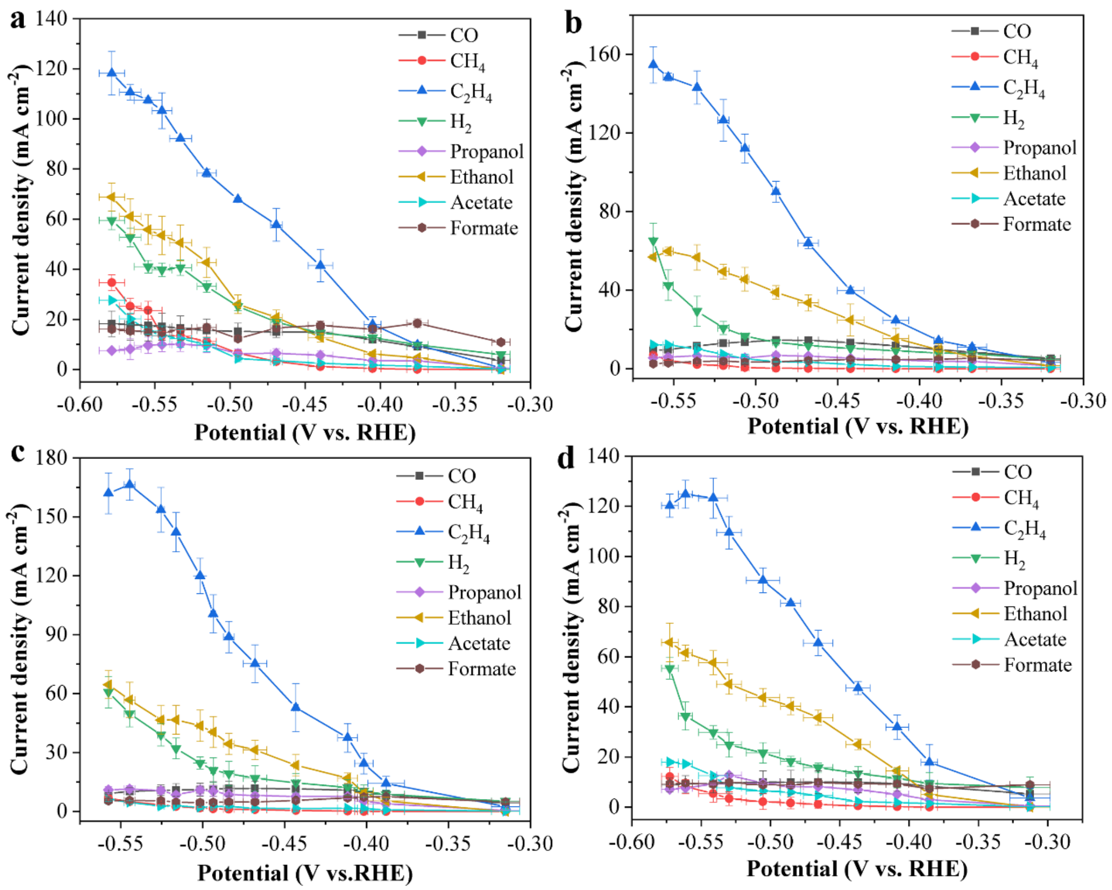

Figure S14. The partial current densities of CO_2 reduction products on CuO-160W electrodes prepared at different electrodeposition current densities. (a) 160 mA cm^{-2}, (b) 180 mA cm^{-2}, (c) 200 mA cm^{-2}, and (d) 220 mA cm^{-2}.

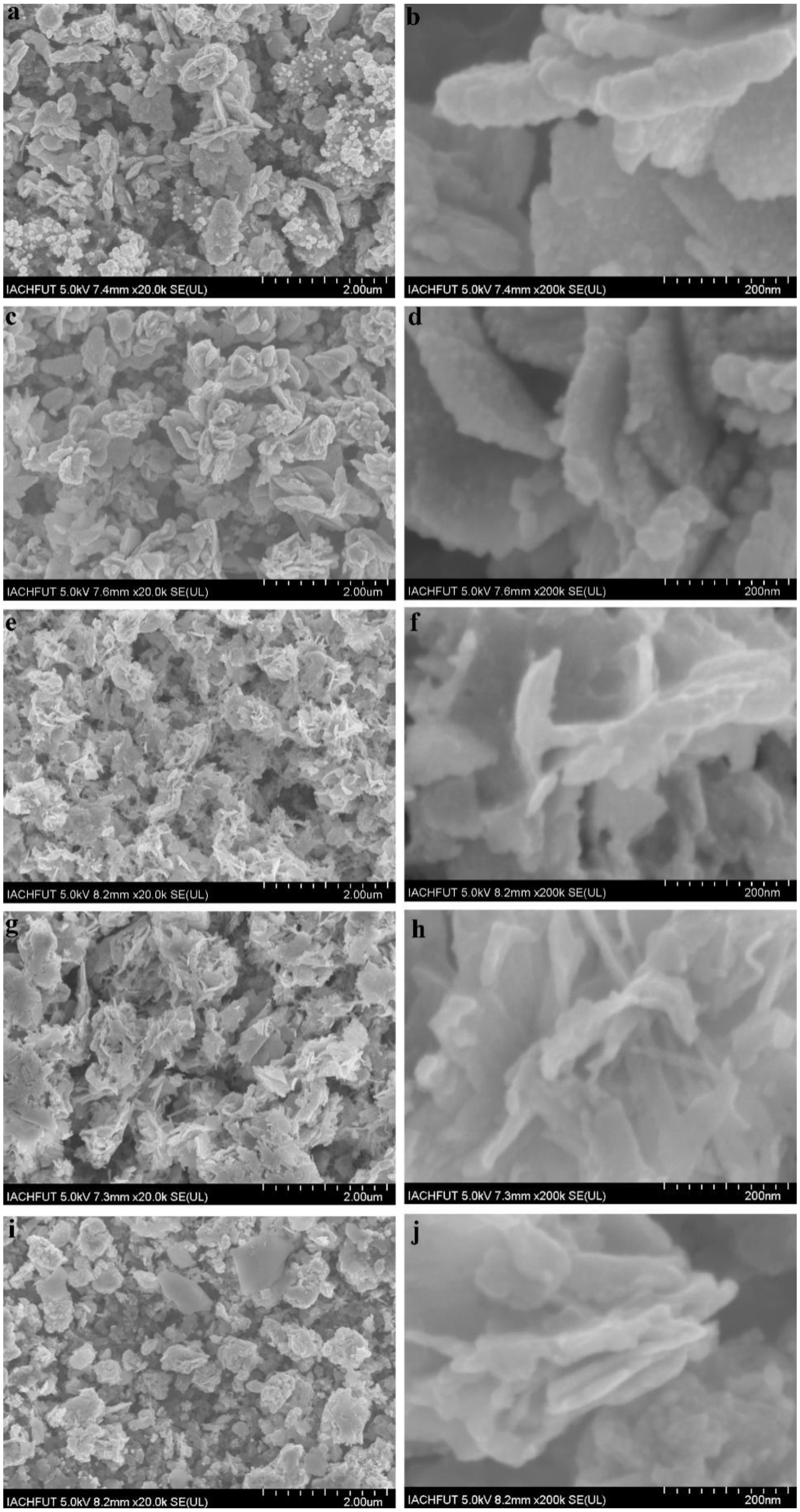
a
IACHFUT 5.0kV 7.4mm x20.0k SE(UL) 2.00um
b
IACHFUT 5.0kV 7.4mm x200k SE(UL) 200nm
c
IACHFUT 5.0kV 7.6mm x20.0k SE(UL) 2.00um
d
IACHFUT 5.0kV 7.6mm x200k SE(UL) 200nm
e
IACHFUT 5.0kV 8.2mm x20.0k SE(UL) 2.00um
f
IACHFUT 5.0kV 8.2mm x200k SE(UL) 200nm
g
IACHFUT 5.0kV 7.3mm x20.0k SE(UL) 2.00um
h
IACHFUT 5.0kV 7.3mm x200k SE(UL) 200nm
i
IACHFUT 5.0kV 8.2mm x20.0k SE(UL) 2.00um
j
IACHFUT 5.0kV 8.2mm x200k SE(UL) 200nm

Figure S15. SEM images of various CuO-160W catalysts prepared by different electrodeposition current density after eCO_2RR. (a,b) 160 mA cm^{-2}, (c,d) 180 mA cm^{-2}, (e,f) 190 mA cm^{-2}, (g,h) 200 mA cm^{-2}, and (i, j) 220 mA cm^{-2}.

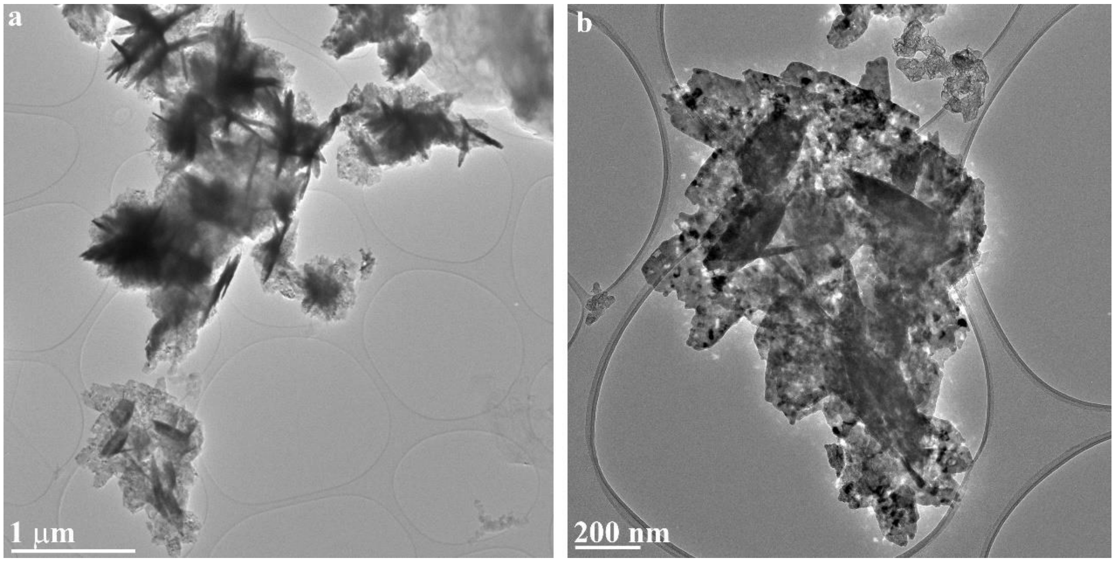

Figure S16. TEM images of CuO-160W nanosheets after eCO_2RR.

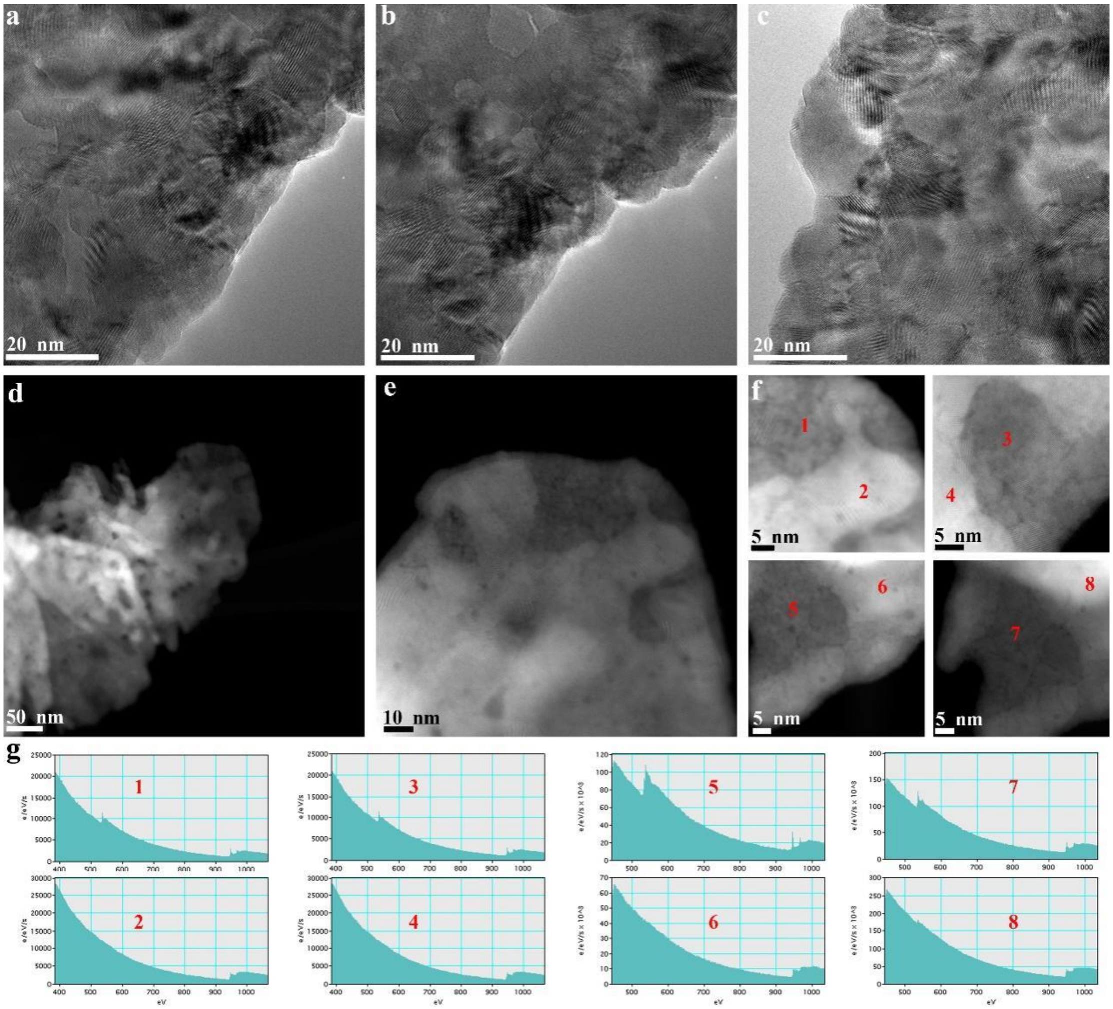

Figure S17. Morphological and composition analysis of post CuO-160W after eCO_2RR. (a-c) HRTEM images, (d-f) HAADF-STEM images, and (g) Corresponding EELS spectra collected from the areas of 1-8 in f.

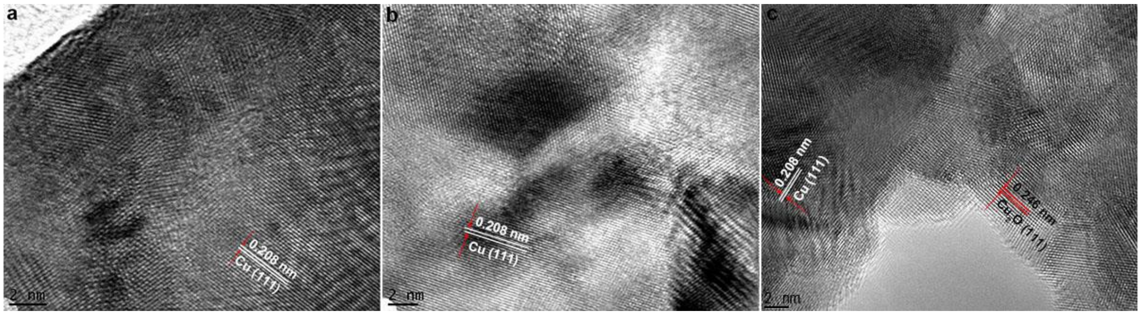

Figure S18. HRTEM images of different post CuO samples after eCO_2RR. (a) CuO-0W, (b) CuO-80W, and (c) CuO-160W.

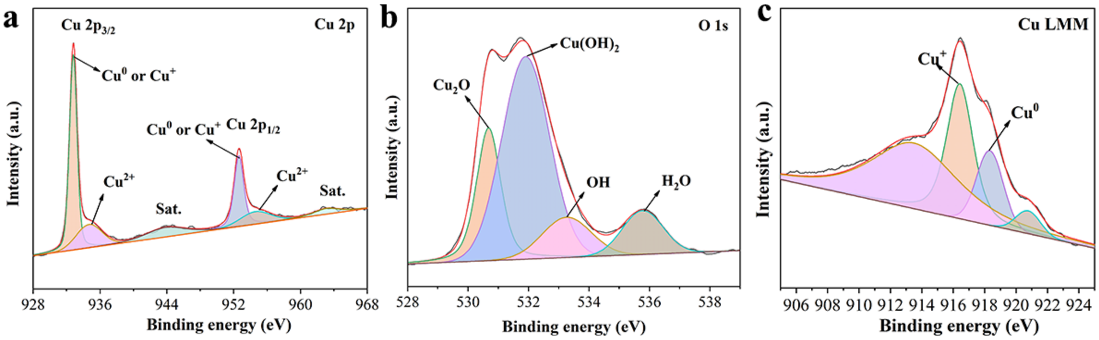

Figure S19. XPS spectra of CuO-160W nanosheets after eCO_2RR. (a) Cu 2p, (b) O1s, and (c) Cu LMM.

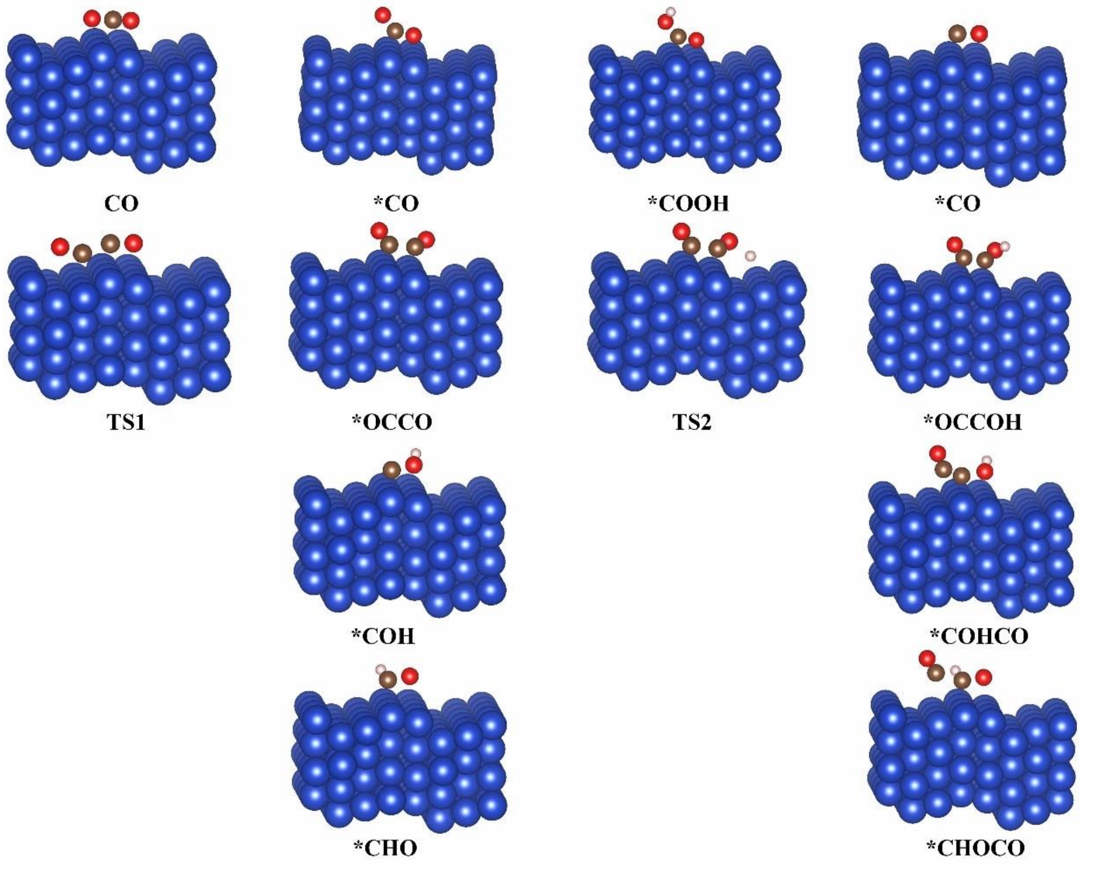

Figure S20. Optimized structures for main reaction intermediates on Cu GBs. The blue, grey, red, and white balls represent Cu, O, C and H, respectively.

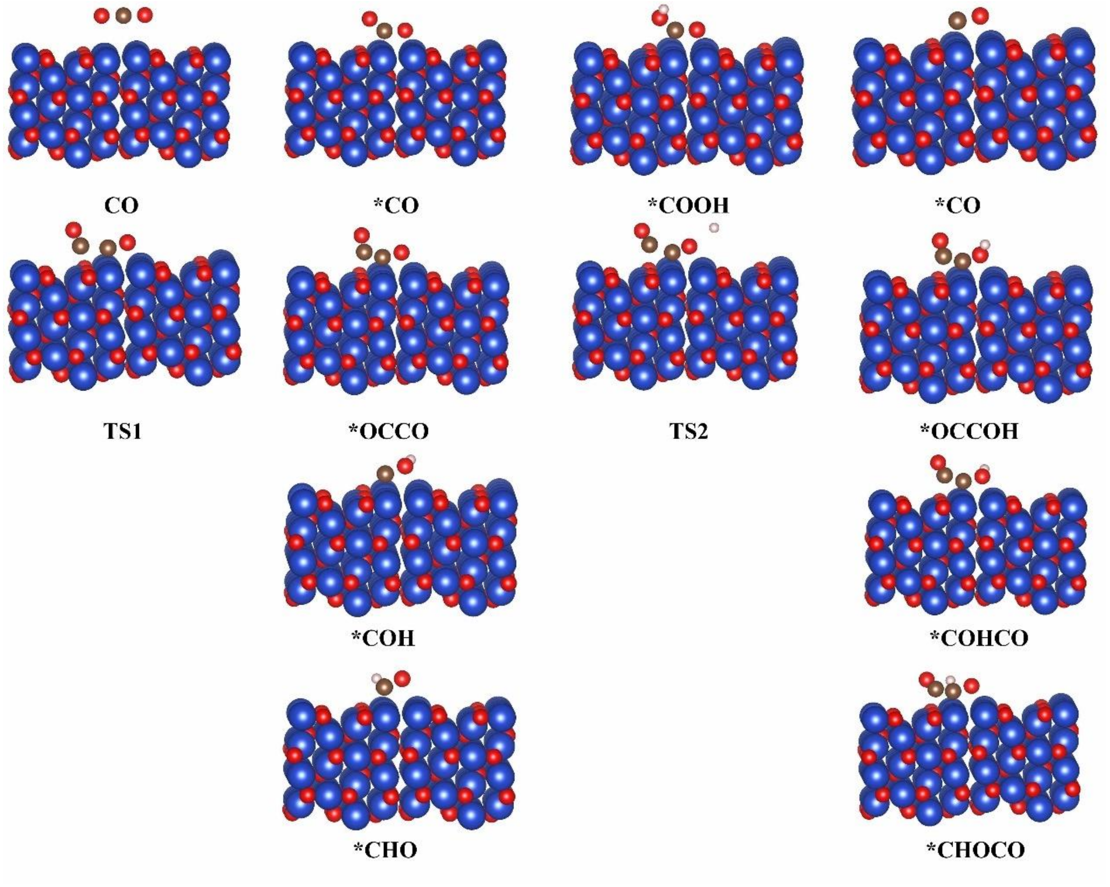

Figure S21. Optimized structures for main reaction intermediates on Cu_2O GBs. The blue, grey, red, and white balls represent Cu, O, C and H, respectively.

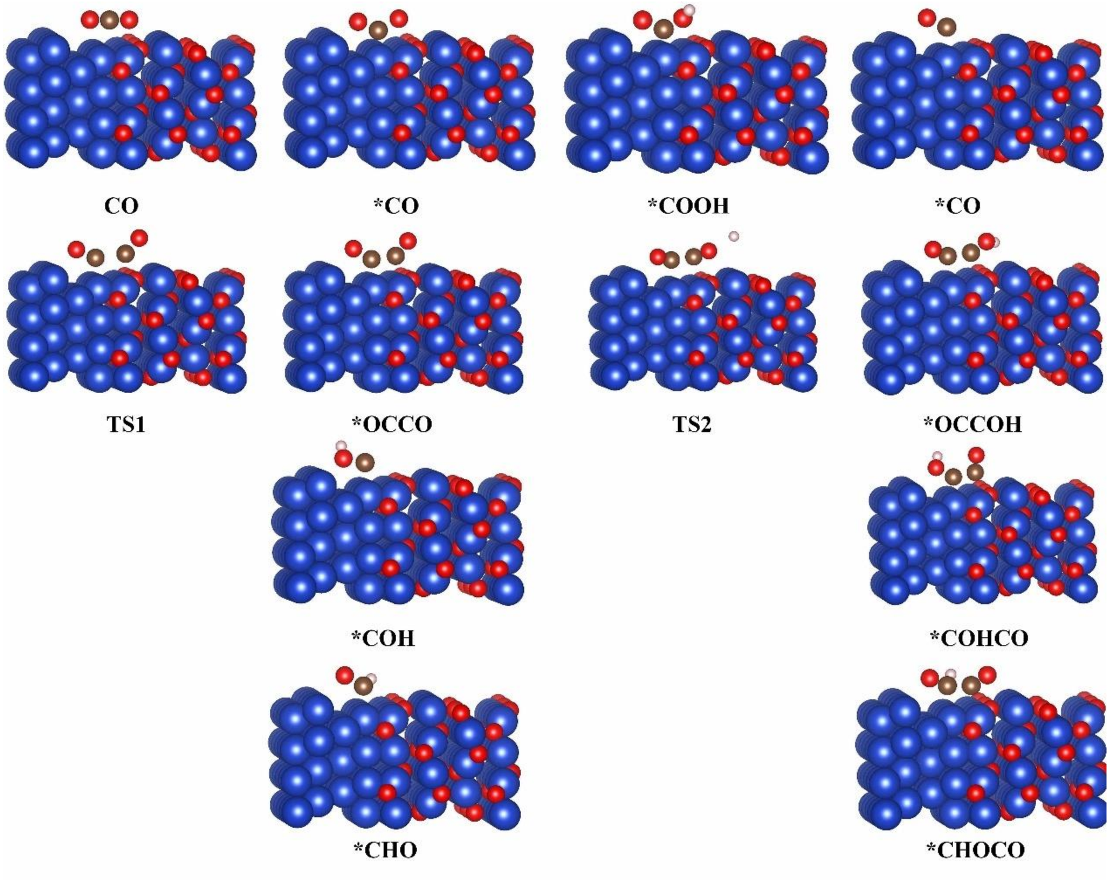

Figure S22. Optimized structures for main reaction intermediates on Cu^{+}/Cu^{0} interfaces. The blue, grey, red, and white balls represent Cu, O, C and H, respectively.

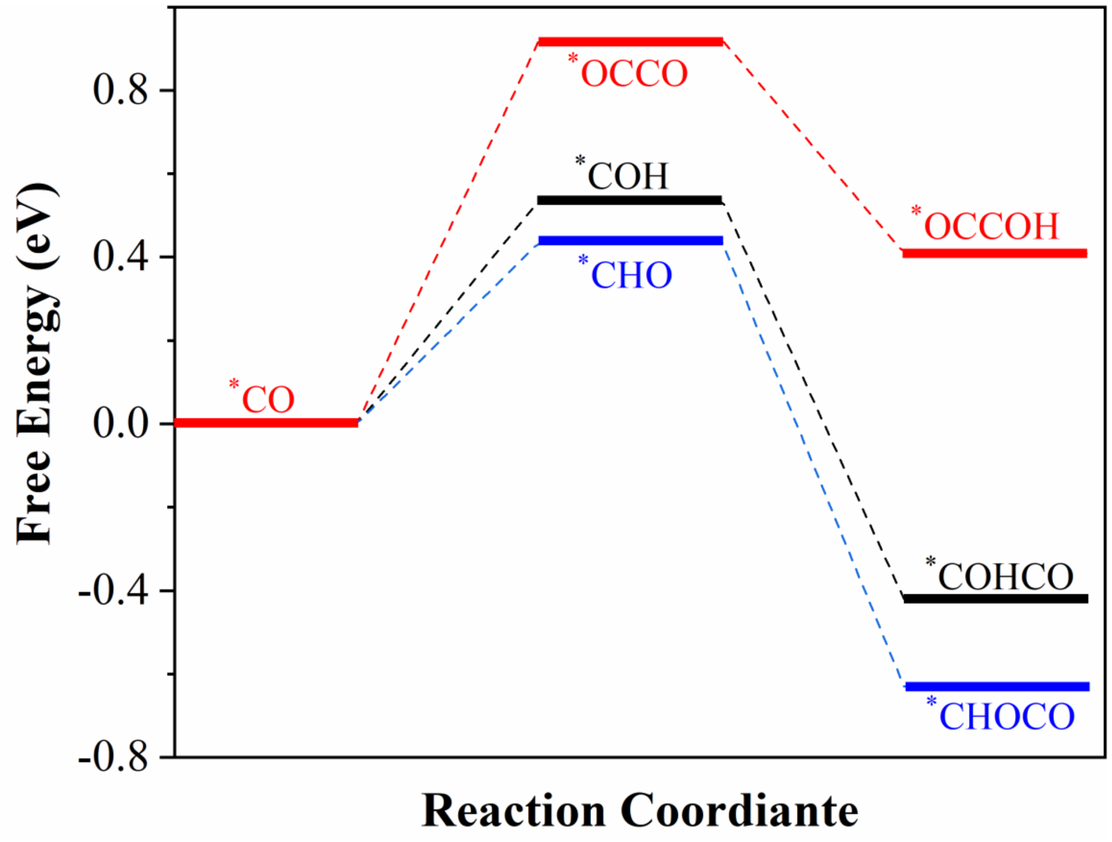

Figure S23. Free energy profiles for the *OCCO, *COH, and *CHO pathway on Cu GBs.

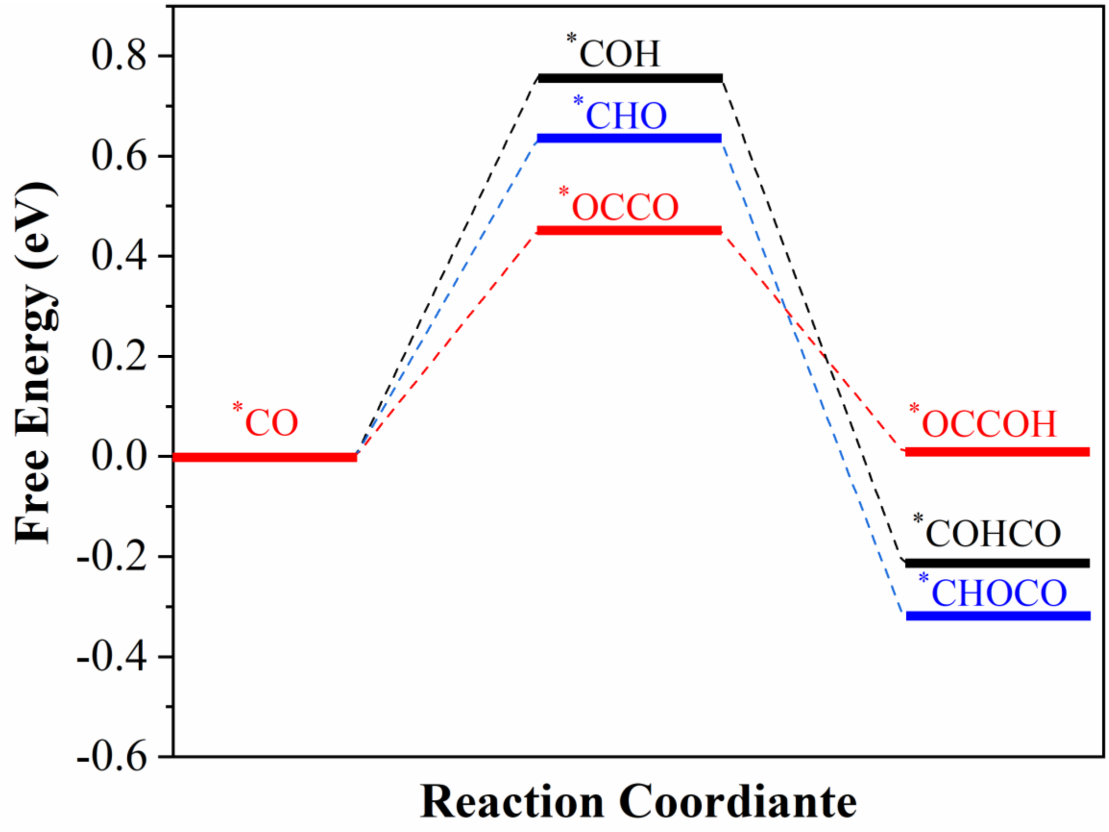

Figure S24. Free energy profiles for the *OCCO, *COH, and *CHO pathway on Cu_2O GBs.

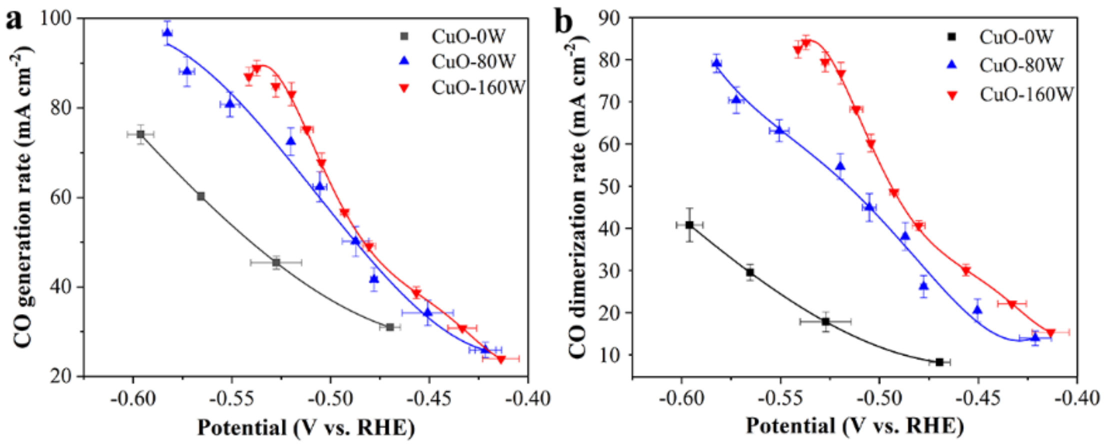

Figure S25. (a) CO generation rates and (b) CO dimerization rates of CuO-0W, CuO-80W, and CuO-160W electrodes under various potentials.

The CO generation rate was defined as the summary of the production rates of C_{2+} products, CH_4 and CO gas products and calculated by the following equation:

$$j_{CO,\ generation} = j_{CO} + \frac{j_{CH4}}{4} + \frac{j_{C2H4}}{3} + \frac{j_{C2H5OH}}{3} + \frac{j_{C3H7OH}}{3} + \frac{j_{CH3COOH}}{2}$$

The CO dimerization was normalized from the production rates of C_{2+} products.

$$j_{CO,\ dimerization} = \frac{j_{C2H4}}{3} + \frac{j_{C2H5OH}}{3} + \frac{j_{C3H7OH}}{3} + \frac{j_{CH3COOH}}{2}$$

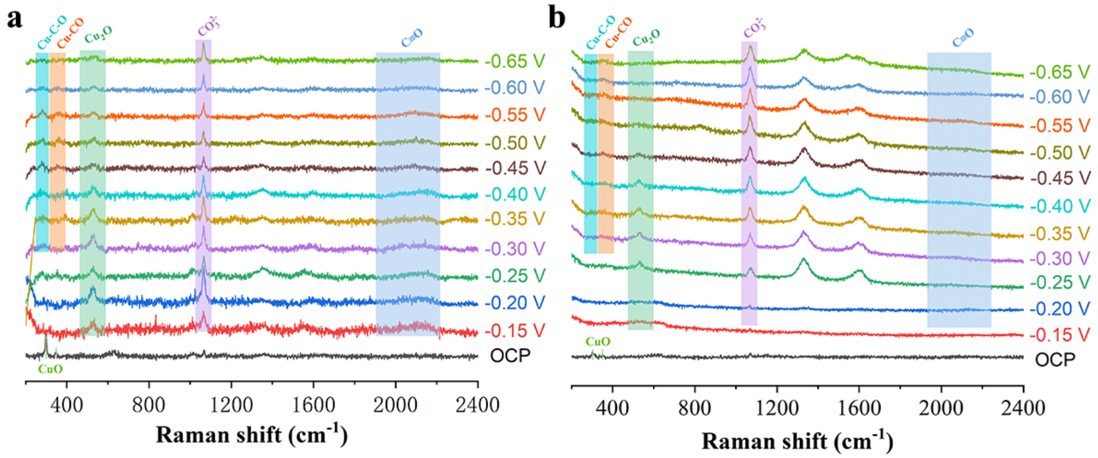

Figure S26. *In-situ* Raman spectra of (a) CuO-80W and (b) CuO-0W electrodes under different potentials for 10 min at each potential

Table S1. The comparison of performance among various Cu-based catalysts for CO_2 reduction to C_2H_4.

Catalyst	electrolyte	Potential (V)	FE_{C2H4} (%)	j_{C2H4} (mA cm^{-2})	CEE_{C2H4} (%)	Ref.
CuO nanosheets	1.0 M KOH	-0.52	62.5	173	41	This work
Cu-Al alloy	1.0 M KOH	-1.5	80	400	34	1
molecules-Cu-12	1.0 M $KHCO_3$	-0.83	72	230	40	2
polyamine-Cu	1.0 M KOH	-0.97	72	312	37	3
Cu-CO_2	7.0 M KOH	-0.67	70	217	42	4
CuAg wires	1.0 M KOH	-0.70	60	180	27	5
Cu hollow spheres	1.0 M KOH	-0.9	61.1	143	33	6
Cu nanoparticles	1.0 M KOH	-0.78	46	150	26	7
Dendritic Copper	0.1 M KBr	-1.1	57	170	28	8
CuDAT wire	1.0 M KOH	-0.6	40	78	25	9
Cu_2O nanoparticles	0.1 M $KHCO_3$	-1.1	57.3	11	28	10
Cu nanosheets	0.1 M K_2SO_4	-1.18	83.2	66.5	39	11
Cu nanoparticles	0.1 M $KHCO_3$	-1.1	45	15.7	22	12
plasma activated Cu	0.1 M $KHCO_3$	-0.9	60	12	32	13

References:

[1] M. Zhong, K. Tran, Y. Min, C. Wang, Z. Wang, C. T. Dinh, P. De Luna, Z. Yu, A. S. Rasouli, P. Brodersen, S. Sun, O. Voznyy, C. S. Tan, M. Askerka, F. Che, M. Liu, A. Seifitokaldani, Y. Pang, S. C. Lo, A. Ip, Z. Ulissi, E. H. Sargent, *Nature* **2020**, *581*, 178.

[2] F. Li, A. Thevenon, A. Rosas-Hernandez, Z. Wang, Y. Li, C. M. Gabardo, A. Ozden, C. T. Dinh, J. Li, Y. Wang, J. P. Edwards, Y. Xu, C. McCallum, L. Tao, Z. Q. Liang, M. Luo, X. Wang, H. Li, C. P. O'Brien, C. S. Tan, D. H. Nam, R. Quintero-Bermudez, T. T. Zhuang, Y. C. Li, Z. Han, R. D. Britt, D. Sinton, T. Agapie, J. C. Peters, E. H. Sargent, *Nature* **2020**, *577*, 509.

[3] X. Chen, J. Chen, N. M. Alghoraibi, D. A. Henckel, R. Zhang, U. O. Nwabara, K. E. Madsen, P. J. A. Kenis, S. C. Zimmerman, A. A. Gewirth, *Nat. Catal.* **2020**, *4*, 20.

[4] Y. Wang, Z. Wang, C.-T. Dinh, J. Li, A. Ozden, M. Golam Kibria, A. Seifitokaldani, C.-S. Tan, C. M. Gabardo, M. Luo, H. Zhou, F. Li, Y. Lum, C. McCallum, Y. Xu, M. Liu, A. Proppe, A. Johnston, P.

Todorovic, T.-T. Zhuang, D. Sinton, S. O. Kelley, E. H. Sargent, *Nat. Catal.* **2019**, *3*, 98.

[5] T. T. H. Hoang, S. Verma, S. Ma, T. T. Fister, J. Timoshenko, A. I. Frenkel, P. J. A. Kenis, A. A. Gewirth, *J. Am. Chem. Soc*. **2018**, *140*, 5791.

[6] R.-X. Yang, Y.-R. Wang, G.-K. Gao, L. Chen, Y. Chen, S.-L. Li, Y.-Q. Lan, *Small Struct*. **2021**, *2*, 2100012.

[7] S. Ma, M. Sadakiyo, R. Luo, M. Heima, M. Yamauchi, P. J. A. Kenis, *J. Power Sources* **2016**, *301*, 219.

[8] C. Reller, R. Krause, E. Volkova, B. Schmid, S. Neubauer, A. Rucki, M. Schuster, G. Schmid, *Adv. Energy Mater.* **2017**, *7*, 1602114.

[9] T. T. H. Hoang, S. Ma, J. I. Gold, P. J. A. Kenis, A. A. Gewirth, *ACS Catal*. **2017**, *7*, 3313.

[10] H. Jung, S. Y. Lee, C. W. Lee, M. K. Cho, D. H. Won, C. Kim, H. S. Oh, B. K. Min, Y. J. Hwang, *J. Am. Chem. Soc.* **2019**, *141*, 4624.

[11] B. Zhang, J. Zhang, M. Hua, Q. Wan, Z. Su, X. Tan, L. Liu, F. Zhang, G. Chen, D. Tan, X. Cheng, B. Han, L. Zheng, G. Mo, *J. Am. Chem. Soc.* **2020**, *142*, 13606.

[12] Z. Xu, T. Wu, Y. Cao, C. Chen, X. Zeng, P. Lin, W.-W. Zhao, *J. Catal*. **2020**, *383*, 42.

[13] H. Mistry, A. S. Varela, C. S. Bonifacio, I. Zegkinoglou, I. Sinev, Y. W. Choi, K. Kisslinger, E. A. Stach, J. C. Yang, P. Strasser, B. R. Cuenya, *Nat. Commun*. **2016**, *7*, 12123.
